# Nonlinear FOPID controller design for pressure regulation of steam condenser via improved metaheuristic algorithm

**DOI:** 10.1371/journal.pone.0309211

**Published:** 2024-09-19

**Authors:** Sarah A. Alzakari, Davut Izci, Serdar Ekinci, Amel Ali Alhussan, Fatma A. Hashim

**Affiliations:** 1 Department of Computer Sciences, College of Computer and Information Sciences, Princess Nourah bint Abdulrahman University, Riyadh, Saudi Arabia; 2 Department of Computer Engineering, Batman University, Batman, Turkey; 3 Applied Science Research Center, Applied Science Private University, Amman, Jordan; 4 Faculty of Engineering, Helwan University, Helwan, Egypt; 5 MEU Research Unit, Middle East University, Amman, Jordan; Chandigarh University, INDIA

## Abstract

Shell and tube heat exchangers are pivotal for efficient heat transfer in various industrial processes. Effective control of these structures is essential for optimizing energy usage and ensuring industrial system reliability. In this regard, this study focuses on adopting a fractional-order proportional-integral-derivative (FOPID) controller for efficient control of shell and tube heat exchanger. The novelty of this work lies in the utilization of an enhanced version of cooperation search algorithm (CSA) for FOPID controller tuning, offering a novel approach to optimization. The enhanced optimizer (en-CSA) integrates a control randomization operator, linear transfer function, and adaptive p-best mutation integrated with original CSA. Through rigorous testing on CEC2020 benchmark functions, en-CSA demonstrates robust performance, surpassing other optimization algorithms. Specifically, en-CSA achieves an average convergence rate improvement of 23% and an enhancement in solution accuracy by 17% compared to standard CSAs. Subsequently, en-CSA is applied to optimize the FOPID controller for steam condenser pressure regulation, a crucial aspect of heat exchanger operation. Nonlinear comparative analysis with contemporary optimization algorithms confirms en-CSA’s superiority, achieving up to 11% faster settling time and up to 55% reduced overshooting. Additionally, en-CSA improves the steady-state error by 8% and enhances the overall stability margin by 12%.

## Introduction

Shell and tube heat exchangers play a pivotal role in various industrial processes, serving as vital components for efficient heat transfer between fluid streams [1]. The effective operation of these heat exchangers is crucial for optimizing energy usage and ensuring the reliability of industrial systems. Controlling the performance of shell and tube heat exchangers has become a critical aspect of industrial processes, as it directly impacts overall efficiency, energy consumption, and system stability [2–4].

The development of advanced control methodologies for optimizing the operation of shell and tube heat exchangers has received considerable attention in recent years. Researchers have explored various control strategies to enhance the dynamic response, robustness, and adaptability of these structures. Several studies have investigated the application of different techniques, striving to address the unique challenges posed by the dynamic nature of heat exchanger systems [5–10].

Recent literature highlights several advanced approaches in this domain. For instance, Li and Wang [11] presented a dynamic model of a steam condenser and proposed a proportional-integral (PI) controller designed using the grey wolf optimizer. This work laid the foundation for employing nature-inspired algorithms in the context of heat exchanger control. Following up on this, Wang and Li [12] looked into the use of optimization algorithms even more. They used particle swarm optimization to create a proportional-integral-derivative (PID) controller for a steam condenser. This study demonstrated the effectiveness of swarm intelligence in tuning controllers for heat exchangers. Reddy and Balaji [13] introduced a genetic algorithm based PID controller specifically for temperature control in shell and tube heat exchangers. The study emphasized the significance of genetic algorithms in optimizing PID parameters for enhanced temperature regulation. Olana and Abose [14] did another study on PID temperature controller design for shell and tube heat exchangers. This study provided additional insights into the application of traditional PID control strategies in this field. Suthar and Gadit [15] explored a two-degree-of-freedom controller optimization using genetic algorithms for shell and tube heat exchangers. This work introduced a more advanced control structure, emphasizing the importance of multi-variable control strategies. Tugashova and Zatonskiy [16] undertook a comprehensive comparison of various control methods for heat exchangers, shedding light on the relative merits of different approaches. Al-Dhaifallah [17] introduced fuzzy fractional-order PID control as an innovative approach for heat exchanger control, demonstrating the integration of fuzzy logic and fractional-order control in this application. Oravec et al. [18] delved into the robust model predictive control and PID control of shell-and-tube heat exchangers. Their work provided insights into the robustness and predictive capabilities of advanced control methodologies in heat exchanger applications. Girirajan and Rathikarani [19] suggested a meta-heuristic optimization algorithm-based optimal CRONE controller. This helped researchers look into more advanced control structures for shell and tube heat exchangers. Ahn et al. [20] incorporated feedforward control and anti-windup techniques in the PID control of a shell and tube heat exchanger system. This work highlighted the importance of integrating additional control features for improved performance. The study in [21] provides a thorough review of methods for enhancing heat transfer in shell and tube heat exchangers by highlighting the importance of passive methods, such as air injection and nanofluids, in improving heat transfer efficiency. The study identifies a need for further research on combining passive methods, geometric modifications to tube surfaces, and theoretical analysis to advance heat transfer enhancement in shell and tube heat exchangers.

Despite these advancements, there are still several research gaps and limitations in the existing studies. Many of the current methods, while innovative, may struggle with issues such as computational complexity, slow convergence rates, and limited adaptability to varying operational conditions. Additionally, the integration of more sophisticated optimization techniques with traditional control methods has not been extensively explored

This study aims to address these gaps by introducing an enhanced cooperation search algorithm (en-CSA) for tuning a fractional-order proportional-integral-derivative (FOPID) controller specifically for shell and tube heat exchangers. The use of FOPID controllers in heat exchangers can impact efficiency by offering better tuning knob systems and increased parameters for tuning. FOPID controllers are more reliable and useful compared to typical PID controllers, providing more efficient and reliable software solutions for complex implementations [22–28]. In this regard, FOPID controllers have been shown to outperform other control strategies in various applications such as wind turbine generators, twin rotor systems, and rail vehicle tilt control [29, 30]. However, there are concerns about implementation complexity and cost, which need to be weighed against the benefits of additional tuning flexibility [31]. In this context, the tuning of the FOPID controller becomes a critical aspect of ensuring its optimal performance. In response to these challenges, this study introduces en-CSA, a novel tuning mechanism utilizing an enhanced version of the cooperation search algorithm (CSA) [32], aiming to improve the efficiency and adaptability of shell and tube heat exchangers.

The original CSA exhibits limitations, such as the inability to ensure a balanced transfer between exploration and exploitation, leading to local optima, a fixed movement step value based on the fitness score across all iterations, constraining global exploration and local exploitation capabilities, and a lack of selection to eliminate undesired search spaces for efficient solution discovery. Therefore, this study proposes en-CSA that uses four main methods to make the original CSA work better: a control randomization operator, a linear transfer function, exploration-exploitation based on adaptive p-best mutation, and a greedy selection strategy. We rigorously tested the en-CSA across the benchmark functions of the CEC2020. We evaluated the en-CSA’s performance in comparison with the original CSA [32], chaos game optimization [33], Harris hawks optimization [34], salp swarm algorithm [35], giant trevally optimizer [36], whale optimization algorithm [37], particle swarm optimization [38], and tree-seed algorithm [39]. The algorithm exhibited robust performance, showcasing its efficacy in optimizing complex functions. Across a diverse set of optimization problems, en-CSA consistently demonstrated competitive convergence rates and accuracy in locating optimal solutions. The algorithm was better at balancing exploration and exploitation thanks to new features like the control randomization operator, linear transfer function, and adaptive p-best mutation. This helped it perform well on the challenging CEC2020 benchmark functions. The achieved results underscore the potential of en-CSA as a versatile and powerful optimization tool, poised to tackle real-world problems with complex and dynamic solution spaces.

The use of en-CSA to tune the FOPID controller in the shell and tube heat exchanger yielded promising results. This research looked at how the Runge-Kutta optimizer [40], prairie dog optimization [41], and RIME optimizer [42] compared to other modern and useful optimization algorithms. The FOPID controller, optimized using en-CSA, demonstrated superior performance in regulating pressure within the heat exchanger system. The en-CSA-tuned FOPID exhibited enhanced control precision, faster response times, and minimized overshooting, showcasing its effectiveness in achieving optimal pressure regulation. The new tuning mechanism in en-CSA made it easy to change the FOPID parameters so that they fit the changing properties of the steam condenser. This made sure that the control would work well even when the operating conditions changed. In the context of comparing the performance of the recommended en-CSA with established approaches reported in the literature, reported approaches such as grey wolf optimizer, particle swarm optimization, genetic algorithm, and Ziegler-Nichols method [11] were also adopted. The application of en-CSA to FOPID controller tuning in steam condenser pressure regulation shows significant improvements, achieving up to 11% faster response times and up to 55% reduced overshooting compared to alternative methods. This study’s contributions lie in the development and application of a highly effective optimization algorithm for improving the control and operational stability of shell and tube heat exchangers, thereby enhancing overall industrial efficiency.

## Cooperation search algorithm

The cooperation search algorithm (CSA) [32] is a population-based metaheuristic designed to emulate cooperative behavior observed in social communities. CSA is recognized for its versatility in addressing various optimization problems and its potential to deliver high-quality solutions. The algorithm employs a population of agents, each representing a potential solution to the optimization problem. These agents interact and cooperate to explore the search space, aiming to discover optimal or near-optimal solutions.

CSA’s primary components include agent initialization, cooperation, exploitation, and termination criteria. Initially, a population of agents is randomly generated, each characterized by decision variables defining its position in the search space. The cooperation phase is critical, involving information exchange among agents to enhance individual solutions. Cooperative mechanisms, such as sharing best solutions or exchanging search directions, facilitate this exchange, enhancing population diversity. The subsequent exploitation phase refines solutions using the gained knowledge through local search operations, aiming for optimal or near-optimal solutions. CSA iteratively continues cooperation and exploitation until a termination criterion, such as a maximum iteration limit, a specific fitness value, or a computational time limit, is met. Once the termination criterion is satisfied, the algorithm returns the best solution obtained during the search process as the final result.

During teamwork formation stage, Eq (1) initializes all team members randomly. Lead solutions are selected from the initial group based on their performance, forming the external best solution set where *I* is the number of current solutions, $x_{i,j}^{k}$ is the *j*^th^ value of the *i*^th^ solution at *k*^th^ cycle.


$$x_{i,j}^{k}=\varphi (x_{i},x_{j}),\;i\in [1,I],j\in [1,J],k=1$$ (1)

In teamwork cooperation stage, members share knowledge with the chairman, supervisors, and directors, enhancing the algorithm’s performance through communication and knowledge exchange, as shown in Eq (2). The teamwork communication includes three types of knowledge: The chairman’s knowledge C, the knowledge of the board of Directors D, and the board of supervisors’ knowledge S. The chairman is randomly selected from the directors’ board to simulate the rotation process, whereas all the directors and supervisors are given an equal position in computing *D* and *S*.

$$u_{i,j}^{k+1}=X_{i,j}^{k}+C_{i,j}^{k}+D_{i,j}^{k}+S_{i,j}^{k},i\in [1,I],j\in [1,J],k\in [1,K]$$ (2)

$$C_{i,j}^{k}=log\left(\frac{1}{\Phi (0,1)}\right)\cdot (glBest_{indx,j}^{k}-x_{i,j}^{k})$$ (3)

$$D_{i,j}^{k}=\alpha \cdot \phi (0,1)\left[\frac{1}{M}\sum_{m=1}^{M}\;glBest_{m,j}^{k}-X_{i,j}^{k}\right]$$ (4)

$$S_{i,j}^{k}=B\cdot \phi (0,1)\cdot \left[\frac{1}{I}\sum_{i=1}^{I}\;perBest_{i,j}^{k}-X_{i,j}^{k}\right]$$ (5)

where $u_{i,j}^{k+1}$ is the *j*^th^ value of the *i*^*th*^ group solution at the *k*+1^*th*^ iteration. $perBest_{i,j}^{k}$ is the *j*^th^ value of the *i*^th^ personal best-known solution at the *k*^th^ iteration. $glBest_{indx,j}^{k}$ is the *j*^th^ value of the *indx*^*th*^ best-known global solution since the start to the *k*^th^ iteration. Indx is a randomly chosen index from [1,2,…,*M*]. $C_{i,j}^{k}$ represents the knowledge gained from the chairman randomly chosen from the external elite set. $D_{i,j}^{k}$ and $S_{i,j}^{k}$ are the average gained knowledge from M best-known global solutions learned so far, and *I* is the best-known personal solution. *α* and *β* are the learning coefficients to correct the degree of influence of both $D_{i,j}^{k}$ and $S_{i,j}^{k}$.

In the exploitation stage, members refine their solutions using knowledge gained from leaders and their own experiences. This ensures the gradual improvement of the team’s competitiveness which is described below.


$$v_{i,j}^{k+1}=\left\{\begin{array}{ll} r_{ij}^{k+1} & \mathrm{if}\;(u_{ij}^{k+1}\geq c_{j})\\ p_{i,j}^{k+1} & \mathrm{if}\;(u_{i,j}^{k+1}<c_{j}) \end{array}\right),\;i\in [1,I],j\in [1,J],k\in [1,K]$$ (6)

$$r_{ij}^{k+1}=\left\{ \begin{array}{cc} \phi ({\tilde{x}}_{j}+{\underline{x}}_{j}-u_{ij}^{k+1},c_{j}) & \mathrm{if}\;(\left|u_{i,j}^{k+1}-c_{j}|<\phi (0,1)\cdot |{\overset{‾}{x}}_{j}-{\underline{x}}_{j}\right|)\\ \phi (x_{j},{\overset{‾}{x}}_{j}+x_{j}-u_{i,j}^{k+1}) & \mathrm{Otherwise} \end{array}\right.$$ (7)

$$p_{i,j}^{k+1}=\left\{ \begin{array}{c} \phi (c_{j,}{\overset{‾}{x}}_{j}+{\underline{x}}_{j}-u_{i,j}^{k+1})\\ \phi ({\overset{‾}{x}}_{j}+{\underline{x}}_{j}-u_{i,j}^{k+1},{\overset{‾}{x}}_{j}) \end{array}\mathrm{if}\;\left(|u_{i,j}^{k+1}-c_{j}|<\phi (0,1)\cdot |{\overset{‾}{x}}_{j}-{\underline{x}}_{j}|\right)\right.$$ (8)

$$c_{j}=({\overset{‾}{x}}_{j}+{\underline{x}}_{i})\cdot 0.5$$ (9)

Here, $v_{i,j}^{k+1}$ represents the *j*^th^ value of *i*^th^ reflective solution at the (*k*+1)^th^ iteration. The team slowly improves its competitiveness by ensuring the attainment of all the team members with superior performance as follows:

$$x_{i,j}^{k+1}=\left\{ \begin{array}{c} u_{i,j}^{k+1}\;if(F(u_{i}^{k+1})\leq F(\nu_{i}^{k+1}))\\ \nu_{i,j}^{k+1}\;if(F(u_{i}^{k+1})>F(\nu_{i}^{k+1})) \end{array}\right.,i\in [1,I],j\in [1,J],k\in [1,K]$$ (10)

where *F*(*x*) is the fitness value of solution *x*. Many constraints are used, and all the variables in solution *x* are firstly adjusted to the feasible region by Eq (11). Afterward, penalty functions in Eq (12) are utilized to obtain the fitness value F(x) by combining the value of constraint violation and the objective value f(x). Then, all constraints are well established for the feasible solutions so that the fitness value is equal to the objective value. On the other hand, the constraint violation value is set to positive for the infeasible solutions so that the fitness value becomes larger than the objective value. Consequently, the group can be guided toward the feasible search area.

$$x_{j}=max\left\{min\{{\overset{‾}{x}}_{j},x_{j}\},{\underline{x}}_{j}\right\}$$ (11)

$$F(x)=f(x)+\sum_{k=1}^{E}\;c_{e}^{1}\cdot max\{g_{e}(x),0\}+\sum_{f=1}^{E}\;c_{f}^{2}\cdot |h_{f}(x)|$$ (12)

where *x*_*j*_ is the *j*^th^ value of the x solution to be evaluated. $c_{e}^{1}$ and $c_{f}^{2}$ are the coefficients of penalty for the *e*^th^, and the *f*^th^ inequality constraint respectively. Algorithm 1 demonstrates the pseudocode of the CSA.

**Algorithm 1. Pseudocode of CSA**

Define the objective function and the limitations.

Initialization:

 (a) Initialize iteration variable iter = 1. Use Eq (1) to randomly generate the initial swarm in the feasible space.

 (b) Use Eq (12) to determine the fitness values of the initial solutions.

 (c) Set the group and the reflective solutions equal to the initial solution.

End initialization

While iter < = max-iterations do

 For the current swarm do

 Update the best-known personal solution (I)

 Update global best-known solutions found(M)

 Use Eqs (2) – (5), get a set of solutions (I) for global exploitation

 Eqs (6) – (9) are used to get a set of reflective solutions (I) for local exploration

 Eq (12) is used to evaluate the fitness values of the solution sets

 Eq (10) is used in the next iteration to select a set of better solutions (I)

 iter = iter +1

  End for

End while

Set the global best-known solution as the final solution.

## Enhanced cooperation search algorithm

This section introduces the proposed enhanced CSA (en-CSA) optimization method. It commences with an examination of the deficiencies within the original CSA, followed by the development of en-CSA, which enhances the initial population of the problem solution. The CSA algorithm exhibits limitations in the following aspects: (a) Inability to ensure a balanced transfer between exploration and exploitation, leading to local optima, (b) fixed movement step value based on the fitness score across all iterations, constraining global exploration and local exploitation capabilities and (c) lack of selection to eliminate undesired search spaces for efficient solution discovery. Motivated by the CSA limitations, en-CSA incorporates four main approaches to enhance performance: (a) Control randomization operator, (b) linear transfer function, (c) exploration-exploitation based on adaptive p-best mutation and (d) greedy selection strategy.

The en-CSA introduces a control randomization operator (Ran) to facilitate the selection of a new position in the direction of the best solution. This mitigates the risk of being trapped in local optima or premature convergence. The Ran operator is employed to modify Eq (12) as expressed in Eq (13), where Ran is defined by Eq (14).


$$C_{i,j}^{k}=log\left(\frac{1}{\Phi (0,1)}\right)\cdot Ran\cdot (glBest_{indx,j}^{k}-x_{i,j}^{k})$$ (13)

Ran=2⋅rand−1 (14)

A linear transfer function (LTF) is employed to enable the gradual transition from exploration to exploitation, ensuring a balance crucial for algorithmic performance. The LTF, governed by Eq (15), is utilized during the new adaptive p-mutation-based exploration-exploitation phase.


$$LTF=exp\;\left(-\frac{t}{T_{max}}\right)$$ (15)

**Algorithm 2. Pseudocode of en-CSA**

Define the objective function and the limitations.

Initialization:

(a) Initialize iteration variable iter = 1.

(b) Use Eq (1) to generate the initial swarm in the feasible zone randomly.

(c) Use Eq (12) to determine the fitness values of the initial solutions

(d) Set the group and the reflective solutions equal to the initial solution.

End initialization

while iter < = max-iterations do

 for the current swarm do

 Use the adaptive p-best mutation to generate the mutant vector y

 Use Eqs (16)–(18) to generate the mutant vector based on adaptive p-best mutation

 Update the best-known personal solution (I)

 Update global best-knoun solutions found(M)

 Use Eqs (2), (19), (4), (5), to get a set of solutions (I) for global exploitation

 Eqs (6)–(9) are used to get a set of reflective solutions (I) for local exploration

 Eq (12) is used to evaluate the fitness values of the solution sets

 Eq (10) is used in the next iteration to select a set of better solutions (I)

 iter = iter +1

 end for

 Greedy selection for the current and the previous solution sets.

end while

 Set the global best-known solution as the final solution.

The en-CSA also introduces a novel exploration-exploitation strategy based on adaptive p-best mutation. The adaptive p-best mutation algorithm facilitates a gradual transition from exploration to exploitation during iterations. Eq (16) defines the creation of a mutant vector (y), and Eq (17) outlines the linear decreasing rule for adjusting the p-value during the search. The adaptive p-best mutation strategy allows global exploration in early iterations and local exploitation in later iterations, enhancing convergence speed.

$$y=x_{pbest}+F(x_{r2}-x_{r3})$$ (16)

$$p(t)=1-\left(1-\frac{1}{NP}\right)\cdot \frac{t-1}{T_{max}-1}$$ (17)

where *x*_*pbest*_ is randomly chosen from the top solution set *p*×*NP*(*p*∈[0,1]), *t* is the *t*^th^ iteration, *T*_max_ is the number of maximum iterations allowed, *NP* is the number of individuals in the population. Eq (18) presents the mutated vector with the incorporation of LTF for improved exploration-exploitation:

$$y=x_{pbest}+d\cdot F(x_{r2}-x_{r3})\cdot LTF$$ (18)

where *d* is either 1 or -1 based on the relation between the difference vectors *x*_*r*2_ and *x*_*r*3_ as follows:

$$d=\left\{ \begin{array}{cc} 1 & \mathrm{if}\;x_{r2}\;\mathrm{is}\;\mathrm{better}\;\mathrm{than}\;x_{r3}\\ -1 & \mathrm{otherwise} \end{array}\right.$$ (19)

This rule guarantees that the differential variation is oriented toward a better vector, thus increasing the possibility of creating an improved solution. The en-CSA finally employs a greedy selection strategy to choose between generated and current solutions, rejecting poorly generated solutions and maintaining focus on existing promising zones.

## Evaluation of en-CSA performance on CEC2020 functions

### Statistical examination

The efficacy of en-CSA was evaluated using the CEC2020 benchmark functions, which are designed to test optimization algorithms under various complex scenarios. Comparative performance evaluation included the original CSA [32], chaos game optimization (CGO) [33], Harris hawks optimization (HHO) [34], salp swarm algorithm (SSA) [35], giant trevally optimizer (GTO) [36], whale optimization algorithm (WOA) [37], particle swarm optimization (PSO) [38], and tree-seed algorithm (TSA) [39].

Table 1 provides a comprehensive overview of the fitness score results for various algorithms across different CEC2020 benchmark functions. The metrics presented include the minimum (min/best), maximum (max/worst), mean, and standard deviation (Std) of the fitness scores. The en-CSA algorithm consistently demonstrates superior performance, achieving the best mean in eight functions (F2, F3, F4, F5, F6, F7, F8, and F9) and outperforming competitors across all metrics in five functions (F3, F4, F6, F8, and F9).

**Table 1:** The fitness score results of CEC2020 functions.

F-No	Metric	en-CSA	CSA	CGO	HHO	SSA	GTO	WOA	PSO	TSA
F1	min	104.23	109.72	100.00	2911.32	2899.36	102.65	14601681.39	2902.05	2975.94
	max	7647.53	12080.11	262.97	3014.65	3000.26	8330.50	71896902.71	3001.58	5033.48
	mean	1562.72	3614.44	119.25	2982.67	2946.42	1763.22	30458803.92	2958.93	3534.33
	std	1881.67	3899.54	38.34	25.09	32.14	2360.92	16406205.51	31.72	473.86
F2	min	1246.40	1457.84	1775.88	2036.78	2728.18	2122.86	2490.10	1898.96	2764.47
	max	4966.76	2790.72	3674.85	3828.07	5317.92	4191.34	5080.24	3754.47	5704.73
	mean	2066.72	2072.65	2604.48	2898.97	3583.41	3191.16	3744.38	2677.73	4027.50
	std	648.42	380.72	537.34	463.52	592.17	570.34	592.92	464.52	640.75
F3	min	726.93	738.79	1775.88	834.51	738.32	794.15	845.50	741.16	874.44
	max	746.93	798.49	3674.85	970.75	906.72	917.79	1039.34	779.11	1043.47
	mean	733.13	762.19	2604.48	899.98	806.13	841.88	956.97	758.54	949.36
	std	5.21	11.35	537.34	30.12	37.44	30.01	44.85	11.17	41.03
F4	min	1901.29	1900.92	1901.97	1912.75	1902.22	1904.11	1918.40	1901.42	2116.56
	max	1903.87	1908.55	1908.21	1933.42	1908.86	1939.78	2078.22	1905.14	658992.65
	mean	1902.10	1902.99	1904.35	1923.18	1904.72	1912.82	1950.59	1902.61	100756.89
	std	0.58	1.38	1.54	5.26	1.69	7.75	36.95	0.89	167125.85
F5	min	76159.99	75435.33	2341.61	45729.05	10927.14	2609.78	71965.40	19502.83	99730.13
	max	307907.05	299385.88	5788.28	2205721.69	692871.84	18660.45	5026620.80	259007.90	4890611.30
	mean	191047.75	128187.57	3344.23	581330.87	205147.38	6907.76	1196337.82	99603.21	1605778.15
	std	69547.46	40210.67	790.33	447915.40	176172.01	4016.58	1016894.43	60581.07	1355474.71
F6	min	1601.09	1619.32	1608.23	1777.42	1703.15	1613.95	1793.39	1721.37	2147.83
	max	1740.73	2263.50	2326.02	2721.20	2531.58	2510.32	2987.41	2261.98	2794.52
	mean	1622.42	1846.70	1883.58	2161.23	2127.66	1911.57	2422.63	1931.72	2446.01
	std	24.98	160.70	177.11	211.61	212.19	197.50	290.77	131.33	193.34
F7	min	8163.71	5140.66	2355.41	61499.00	6542.34	2543.12	10099.33	4681.22	14109.59
	max	276935.56	329132.69	3338.89	825589.93	527036.09	12731.71	3581768.37	312993.81	11247832.94
	mean	113664.38	97115.46	2835.71	242057.35	88699.02	4308.89	1011533.57	89234.49	1290223.42
	std	76574.61	74965.63	267.02	174732.57	104322.16	1944.63	979760.41	79457.84	2364345.56
F8	min	2300.00	2300.00	2300.00	2311.90	2300.00	2300.00	2317.11	2300.00	2427.88
	max	2302.50	2302.01	2305.59	6081.67	5511.04	5970.91	6726.99	5982.27	6552.63
	mean	2300.48	2300.62	2301.11	4060.82	2907.44	2424.75	3955.57	3197.56	4736.08
	std	0.80	0.70	1.32	1592.76	1066.29	669.77	1696.25	1327.53	1419.33
F9	min	2804.32	2819.01	2814.09	2917.45	2820.62	2836.65	2902.37	2827.72	2950.80
	max	2825.14	2864.53	2928.08	3325.50	2908.91	3031.07	3089.22	2923.88	3238.33
	mean	2812.21	2839.14	2851.55	3109.96	2850.31	2907.49	3000.61	2864.64	3118.54
	std	4.85	12.84	21.10	92.05	22.57	43.73	54.73	25.16	65.14
F10	min	2913.77	2910.41	2910.39	2911.32	2899.36	2903.34	2935.19	2902.05	2975.94
	max	3007.79	3000.75	3005.55	3014.65	3000.26	3013.17	3136.19	3001.58	5033.48
	mean	2980.13	2950.68	2938.33	2982.67	2946.42	2970.87	3036.06	2958.93	3534.33
	std	25.67	34.64	33.47	25.09	32.14	30.42	44.38	31.72	473.86

From a scientific standpoint, the robustness of en-CSA can be attributed to its unique integration of a control randomization operator, linear transfer function, and adaptive p-best mutation, which together enhance the algorithm’s exploration and exploitation capabilities. This balance enables en-CSA to avoid premature convergence and maintain diversity in the search space, which is critical for navigating complex, multimodal landscapes characteristic of the CEC2020 functions.

Table 2 presents the Wilcoxon p-values, indicating the statistical significance of differences between en-CSA and baseline algorithms for each CEC2020 function. P-values below the commonly used significance level of 0.05 suggest significant differences in the performance of en-CSA compared to the respective baseline algorithms. Especially, en-CSA exhibits statistical similarity to the original CSA in functions F2, F7, and F8, underscoring the comparable performance of these two algorithms in these specific functions. These statistical analyses reinforce the reliability and consistency of en-CSA across diverse benchmark functions.

**Table 2:** Wilcoxon p-value of the en-CSA vs baseline algorithms for CEC2020.

	en-CSA vs CSA	en-CSA vs CGO	en-CSA vs HHO	en-CSA vs SSA	en-CSA vs GTO	en-CSA vs WOA	en-CSA vs PSO	en-CSA vs TSA
F1	0.024156885	4.99795E-09	1.28604E-06	2.87897E-06	0.970516051	3.01986E-11	2.00229E-06	4.44405E-07
F2	0.42038633	3.59234E-05	3.96477E-08	5.07231E-10	7.77255E-09	6.12104E-10	1.10772E-06	4.19968E-10
F3	4.97517E-11	3.01986E-11	3.01986E-11	4.50432E-11	3.01986E-11	3.01986E-11	9.91863E-11	3.01986E-11
F4	0.000253058	3.82489E-09	3.01986E-11	8.89099E-10	3.01986E-11	3.01986E-11	0.014412183	3.01986E-11
F5	0.000268057	3.01986E-11	1.16744E-05	0.510597937	3.01986E-11	1.72941E-07	2.67842E-06	2.00229E-06
F6	2.66947E-09	2.0338E-09	3.01986E-11	3.33839E-11	8.89099E-10	3.01986E-11	3.68973E-11	3.01986E-11
F7	0.371077032	3.01986E-11	0.00037704	0.051877131	3.33839E-11	1.86085E-06	0.157975689	0.006972441
F8	0.358204633	0.000473431	2.74777E-11	4.86493E-06	1.28015E-07	2.74777E-11	0.000491767	2.74777E-11
F9	5.49405E-11	9.91863E-11	3.01986E-11	4.97517E-11	3.01986E-11	3.01986E-11	3.01986E-11	3.01986E-11
F10	0.003670893	0.000110577	0.807274951	8.14648E-05	0.283778048	4.11271E-07	0.004225918	1.95678E-10

Table 3 presents the mean ranks resulting from Friedman’s test, which evaluates the overall performance of different algorithms across all CEC2020 functions. The lower the mean rank, the better the performance of the algorithm in this context. The Friedman’s mean rank for en-CSA is notably low at 2.5, indicating its superior performance relative to other algorithms such as CSA, CGO, HHO, SSA, GTO, WOA, PSO, and TSA. This low mean rank suggests that en-CSA consistently achieved better rankings across the CEC2020 functions.

**Table 3:** Mean rank of Friedman’s test for CEC2020.

	En-CSA	CSA	CGO	HHO	SSA	GTO	WOA	PSO	TSA
Friedman’s mean rank	2.5	3.4	3	6.8	4.5	4.3	7.9	4	8.6
General mean rank	1	3	2	7	6	5	8	4	9

Comparing the general mean ranks, en-CSA holds the top position with a rank of 1, reinforcing its overall superiority in performance. This consistent excellence is attributed to the algorithm’s enhanced design, which effectively combines the strengths of various optimization strategies to tackle the intricate challenges posed by the CEC2020 functions.

In-depth analysis of the results from a physical perspective reveals that en-CSA’s superior performance is linked to its ability to manage the trade-off between exploration and exploitation. This balance is crucial for solving high-dimensional and complex optimization problems where global optimality is difficult to achieve. The adaptive mechanisms in en-CSA allow for dynamic adjustments during the optimization process, leading to more precise and stable solutions.

The statistical and physical insights provided by these evaluations underscore the efficacy and robustness of en-CSA in solving complex optimization problems. The enhanced algorithm’s ability to consistently outperform traditional and contemporary optimization techniques highlights its potential for broad applications in various industrial and scientific domains.

### Analysis of convergence behavior

Conducting a convergence analysis for metaheuristic search algorithms entails scrutinizing their behavior over time and monitoring the progression of the algorithm in its pursuit of an optimal solution. This analysis conventionally involves plotting the objective function values across iterations. Convergence curves were generated for the proposed en-CSA and the baseline algorithms applied to the CEC2020 functions.

**Fig 1:**
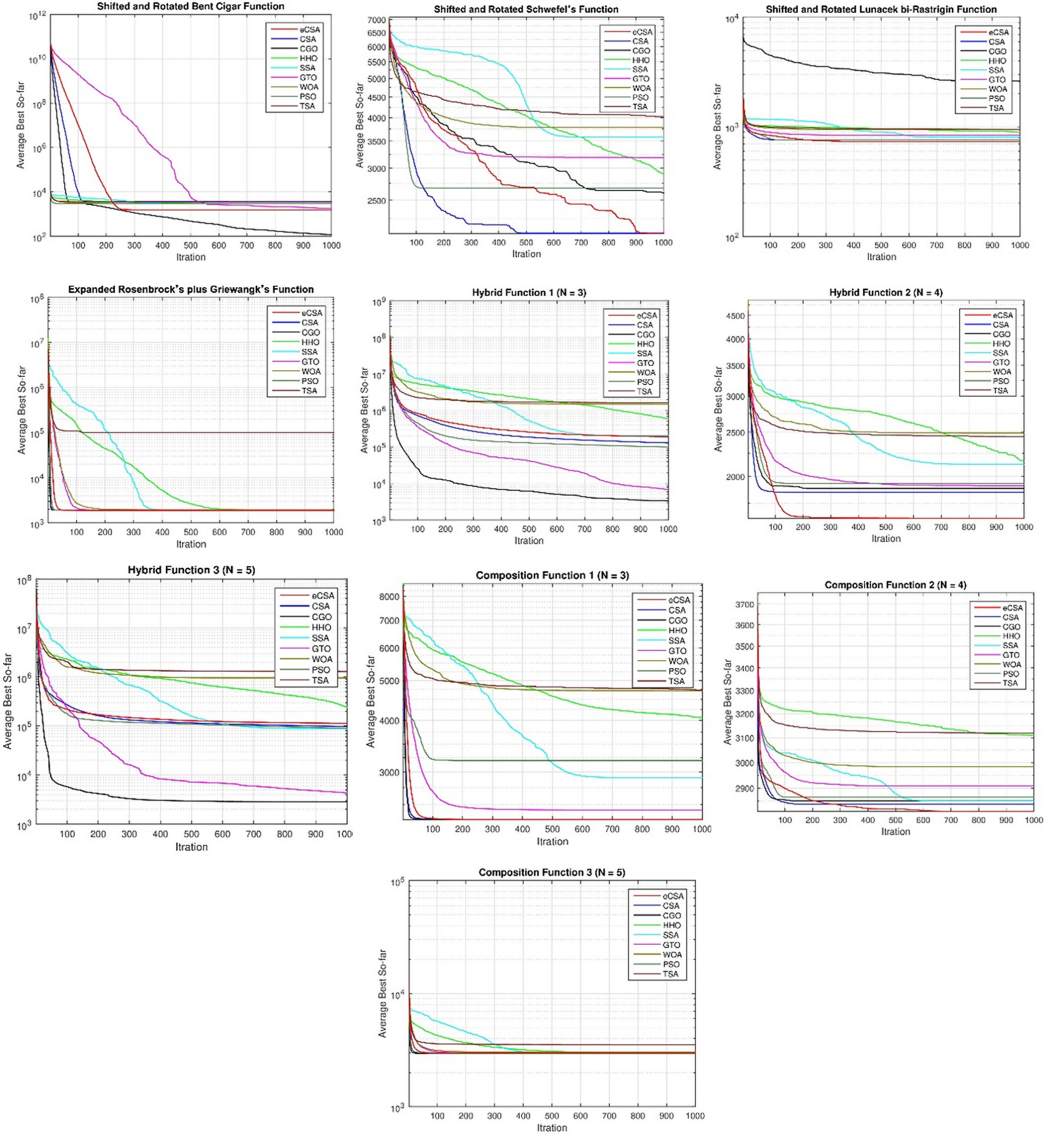
Convergence of en-CSA compared to CSA, CGO, HHO, SSA, GTO, WOA, PSO, and TSA using benchmark functions.

Fig 1 illustrates the convergence curves, depicting the values of the average best for the experimented algorithms at each iteration. The observed curves distinctly showcase that en-CSA attains a superior convergence score compared to its counterparts across all tested functions, except for functions F5 and F7, where CGO exhibits better convergence.

From a scientific standpoint, the convergence behavior of en-CSA can be attributed to its integrated mechanisms—control randomization operator, linear transfer function, and adaptive p-best mutation—that enhance both exploration and exploitation capabilities. These mechanisms allow en-CSA to maintain diversity in the population, preventing premature convergence to local optima and enabling a thorough search of the solution space. This is particularly evident in the convergence curves, where en-CSA consistently progresses towards the global optimum with minimal stagnation.

Analyzing the convergence curves from a physical perspective, the efficiency of en-CSA in navigating the search space can be linked to its ability to dynamically adjust its search parameters based on the landscape of the objective function. In complex, multimodal functions such as those in the CEC2020 benchmark, the landscape often contains numerous local optima that can trap less adaptive algorithms. The control randomization operator in en-CSA introduces a stochastic element that helps in escaping these local optima, while the adaptive p-best mutation fine-tunes the search around promising regions, leading to a more effective and directed search process.

For functions F5 and F7, where CGO exhibits better convergence, it is essential to consider the nature of these specific functions. The superior performance of CGO in these cases may be due to its inherent ability to exploit certain structural characteristics of these functions more effectively. This highlights the importance of understanding the specific problem landscape when choosing or designing optimization algorithms. Nevertheless, this comprehensive analysis substantiates the efficiency of en-CSA in navigating the search space and converging toward optimal solutions for a diverse range of functions within the CEC2020 benchmark. The consistent superior performance of en-CSA, as depicted in the convergence curves, reinforces its effectiveness in optimization endeavors, showcasing its robustness and adaptability in handling complex optimization problems.

### Analysis of boxplot behavior

Boxplots are graphical representations employed to succinctly summarize the distribution of performance scores. This analytical method proves valuable in visually identifying outlier values, assessing the symmetry of the distribution, and comparing both the spread and central tendency of the generated scores. A narrow boxplot indicates a strong correlation between the data points, with a smaller and narrower boxplot being indicative of better performance for an algorithm.

**Fig 2:**
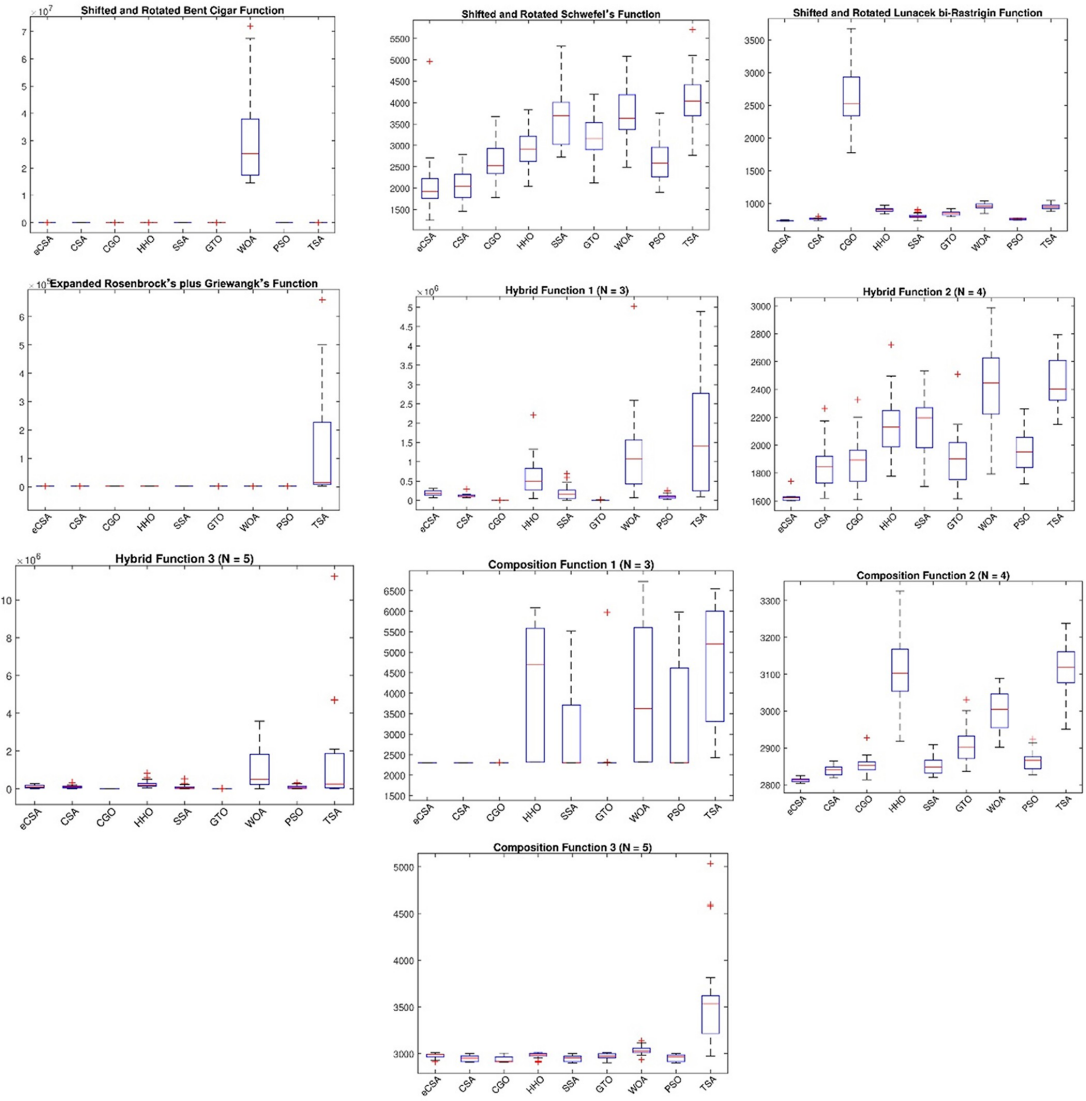
Boxplot of en-CSA compared to CSA, CGO, HHO, SSA, GTO, WOA, PSO, and TSA using benchmark functions.

Fig 2 provides the boxplot curves illustrating the performance of the en-CSA across the CEC2020 functions. The en-CSA boxplots, observed for all tested functions, are notably narrow, suggesting a consistent performance with minimal variability. Additionally, en-CSA consistently exhibits nearly the lowest median and variability among its competitors for each function.

From a scientific standpoint, the narrow and low median boxplots of en-CSA reflect its ability to consistently find high-quality solutions across multiple runs. This consistency is crucial for optimization algorithms as it indicates reliability and robustness in performance. The control randomization operator and adaptive p-best mutation mechanisms in en-CSA contribute to this consistency by ensuring a balanced exploration and exploitation process, reducing the likelihood of being trapped in local optima and enhancing convergence to global optima.

Analyzing the boxplots from a physical perspective, the low variability and median values indicate that en-CSA maintains stable performance even in the presence of complex, multimodal landscapes typical of the CEC2020 functions. The stability is a result of the algorithm’s adaptive mechanisms that dynamically adjust the search strategy based on the landscape of the objective function. This adaptability allows en-CSA to efficiently navigate through different regions of the search space, ensuring that the solutions are not only optimal but also robust against perturbations and variations in the problem landscape.

Moreover, the ability of en-CSA to maintain narrow boxplots across diverse functions highlights its versatility and robustness in handling a wide range of optimization problems. This versatility is particularly important in real-world applications where the nature of the optimization problem can vary significantly, and an algorithm needs to perform well across different scenarios.

This graphical analysis underscores the robust performance and stability of en-CSA across diverse optimization scenarios within the CEC2020 benchmark. The consistently narrow boxplots and low median values reinforce en-CSA’s effectiveness in achieving reliable and consistent results, making it a powerful tool for solving complex optimization problems.

### Exploration–exploitation analysis

**Fig 3:**
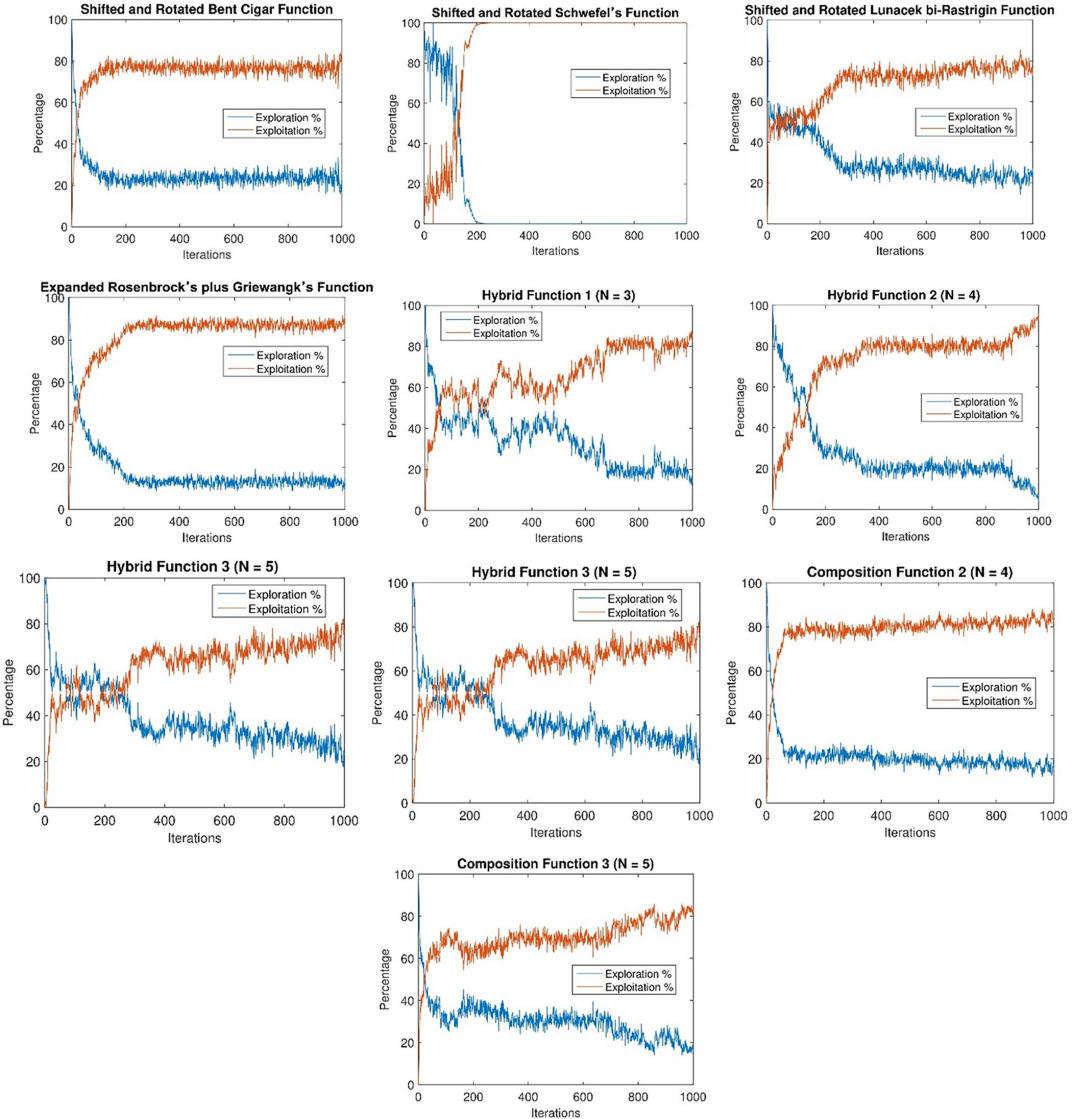
Demonstration of en-CSA’s exploration-exploitation capability through benchmark functions.

The exploration-exploitation dynamics exhibited by en-CSA in addressing the CEC2020 test functions are elucidated in Fig 3. The depicted curves reveal a nuanced and well-balanced exploration-exploitation behavior demonstrated by en-CSA across a majority of CEC2020 functions. Specifically, the algorithm dedicates a substantial amount of time to exploration, particularly during the initial stages of its operation.

From a scientific standpoint, this deliberate emphasis on exploration in the early phases contributes to the algorithm’s efficacy in traversing the solution space comprehensively. Exploration involves investigating diverse regions of the search space to avoid premature convergence to local optima, while exploitation focuses on refining solutions around promising regions to achieve optimality. The control randomization operator and adaptive p-best mutation mechanisms in en-CSA are pivotal in managing this balance. The randomization operator introduces stochasticity, enhancing the exploration capability, while the adaptive p-best mutation fine-tunes the search process, enhancing exploitation.

Analyzing this behavior from a physical perspective, the initial extensive exploration allows en-CSA to build a broad understanding of the solution landscape. This is particularly important for complex, multimodal functions like those in the CEC2020 benchmark, where the landscape may contain numerous local optima. By extensively exploring the search space early on, en-CSA reduces the risk of becoming trapped in suboptimal regions. As the algorithm progresses, it gradually shifts focus towards exploitation, concentrating computational efforts on refining the most promising solutions identified during the exploration phase.

This strategic allocation of computational efforts, as evidenced by the exploration-exploitation curves, underscores en-CSA’s adaptive and effective approach in navigating complex optimization landscapes. The balance between exploration and exploitation is crucial for maintaining diversity in the population and ensuring convergence to high-quality solutions. The dynamic adjustment of this balance by en-CSA, facilitated by its enhanced mechanisms, allows it to adaptively respond to the characteristics of the problem landscape, enhancing its overall optimization performance.

### Analysis of population diversity

**Fig 4:**
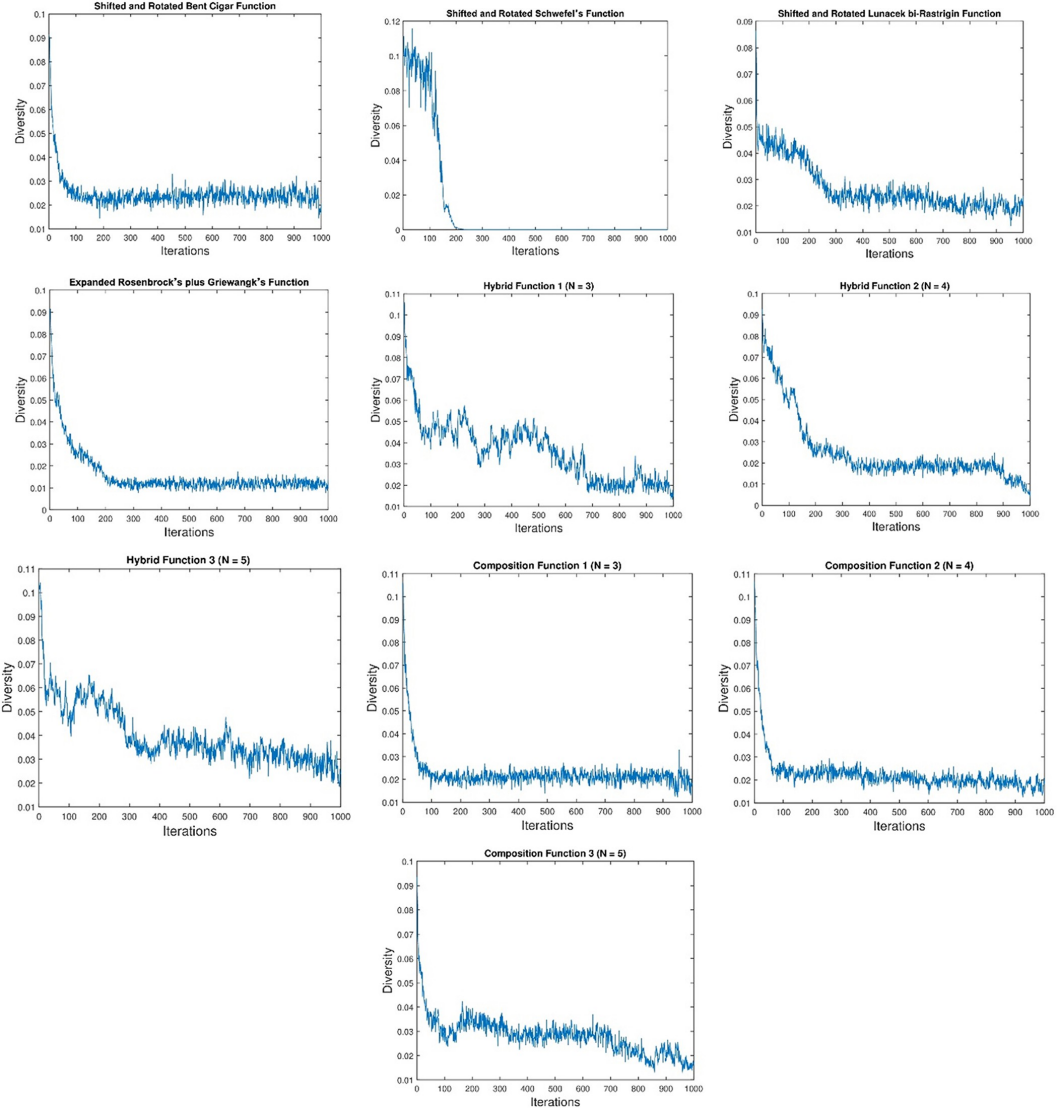
Diversity of the population of en-CSA with respect to benchmark functions.

The examination of population diversity serves as a critical means to assess the equilibrium between exploration and exploitation within the proposed algorithm. In this study, diversity curves of the en-CSA were generated, as depicted in Fig 4. The observed curves delineate that en-CSA consistently upholds heightened population diversity throughout the optimization process.

From a scientific standpoint, maintaining high population diversity is essential for ensuring a comprehensive search of the solution space. High diversity prevents premature convergence to local optima by encouraging the exploration of various regions within the search space. The control randomization operator and adaptive p-best mutation mechanisms in en-CSA play crucial roles in sustaining this diversity. These mechanisms introduce variability and adaptability into the population, allowing the algorithm to continuously generate diverse candidate solutions.

Analyzing the behavior from a physical perspective, the strategic maintenance of diversity is instrumental in mitigating the risk of being confined to local optima, thereby fostering exploration across a broader spectrum of the solution space. This is particularly important for complex, multimodal functions, where the landscape may contain numerous local optima. By maintaining a diverse population, en-CSA ensures that it can explore multiple regions simultaneously, increasing the likelihood of escaping suboptimal solutions and discovering the global optimum.

The deliberate emphasis on increased population diversity augments the algorithm’s capacity for efficient exploitation as well. Once promising regions are identified, the algorithm can effectively refine solutions within these regions, leveraging the diverse candidate solutions to achieve optimal performance. The dynamic adjustment of diversity, facilitated by en-CSA’s enhanced mechanisms, allows the algorithm to balance exploration and exploitation adaptively based on the problem landscape.

This diversity analysis underscores en-CSA’s adaptive and well-informed approach to balancing exploration and exploitation dynamics. The algorithm’s ability to sustain high population diversity throughout the optimization process enhances its robustness and efficacy in navigating complex optimization landscapes. The adaptive mechanisms within en-CSA ensure that it can maintain this balance, thereby contributing to its superior performance in solving the CEC2020 benchmark functions.

## Dynamic modeling of steam condenser

**Fig 5:**
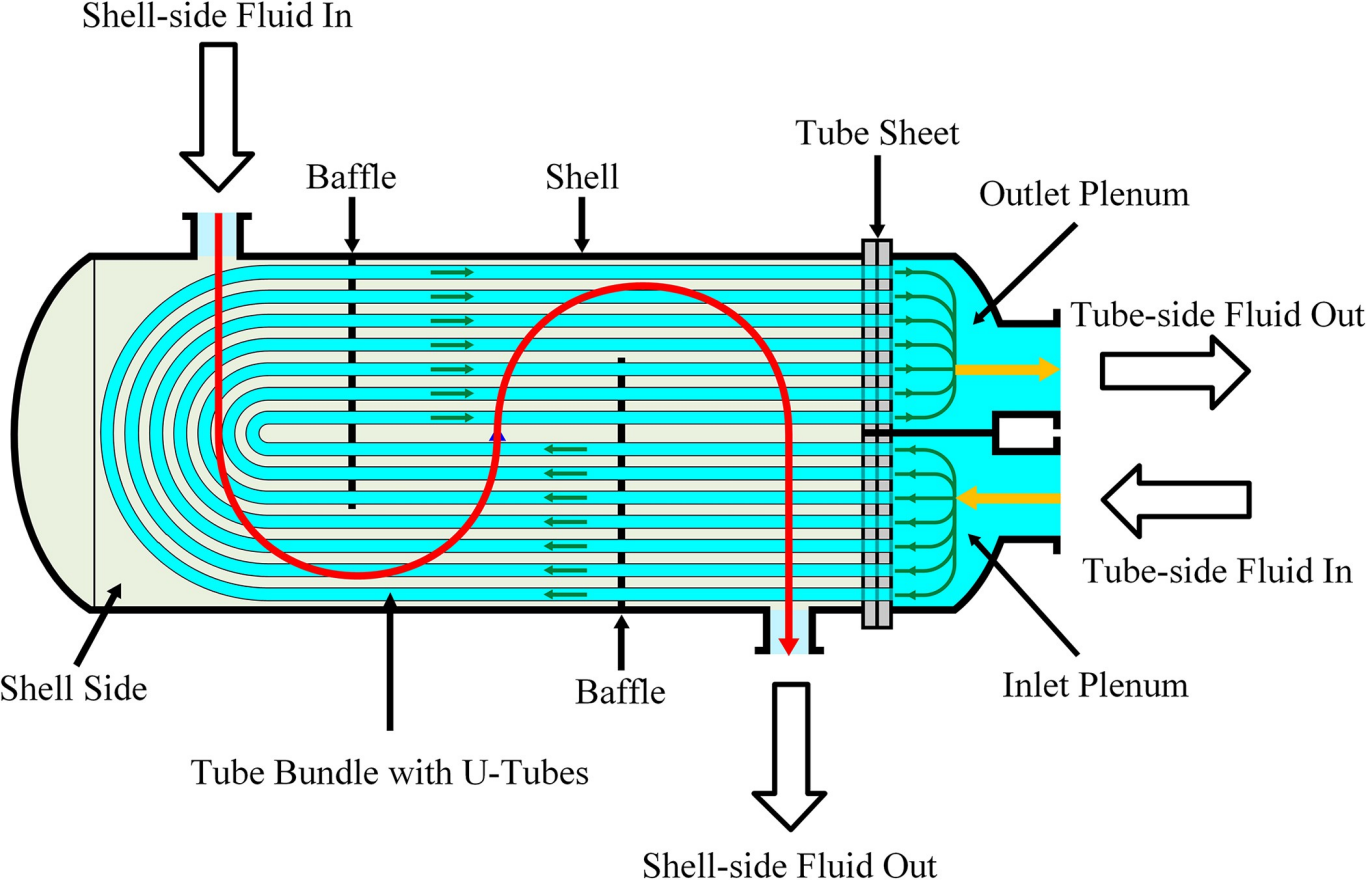
Structure diagram of shell-and-tube condenser.

The outer configuration of a shell-and-tube type condenser typically exhibits a cylindrical or elliptical shape, as illustrated in Fig 5. This structure comprises end closures that form water chambers. A perforated tube plate is positioned between the end closures and the shell, incorporating numerous cooling water pipes arranged hierarchically. Steam enters the condenser shell through an upper steam admission pipe, connected directly or indirectly to exhaust equipment via a compensator. A gathering tank (hot well water tank) for condensed water is located in the lower part of the shell, with the air outlet port situated at its lower section, drawing air through this nozzle.

The operational process of the steam condenser unfolds as follows: Steam is introduced into the condenser via the steam admission pipe, initiating radiant condensation upon contact with the tube wall. Concurrently, latent heat is transferred to the cooling water through the surface of the cooling water pipe. Cooling water, with an inlet temperature, enters the water chamber through the cooling water pipe, distributing across all pipes of the first procedure in the lower part of the condenser shell. The cooling water progresses through subsequent water chambers along the first six cooling water pipes, engaging in heat exchange with the steam. After multiple procedures, the cooling water, now with an outlet temperature, is discharged from the outlet pipes.

Due to the lack of system sealing, air is continuously drawn out from the condenser to maintain the required vacuum degree. The drawn gas consists of both air and steam. Initially, during condensation, the air volume is considerably smaller than the total steam amount. As steam and air move towards the exhaust port, continuous steam condensation leads to a gradual decrease in steam quality within the mixture. Conversely, the relative content of air progressively increases. The steam condensation process concludes when the relative content of air entering the cooling zone reaches a significant level. The mathematical model of the shell-and-tube condenser can be expressed as follows. For the steam zone, the governing equations are provided as [11, 12]:

$$\frac{dGs}{dt}=Gst+Gost-Gc-Gss$$ (20)

$$\frac{dPs}{dt}=\frac{Rs(\frac{dGs}{dt})}{V}(Ts+273.15)$$ (21)

$$\frac{dGsHs}{dt}=Gst\times Hst+Gost\times Host-(Gc+Gss)\times Hs$$ (22)

where *Gs*, *Gst*, *Gost*, *Gc*, *Gss*, *Ps*, *Rs*, *V*, *Ts*, *Hs*, *Hst* and *Host* respectively represent the steam content in the shell side, the exhaust volume of steam turbine, the other steam inlet of the condenser, the main steam condensate, the amount of steam drawn out by vacuum pumping equipment, the internal steam pressure of the condenser, the steam gas constant, volume of gas in the condenser, temperature of the saturated gas, the average enthalpy of the steam, the enthalpy of the steam turbine exhaust, and other inlet enthalpy. Eqs (20), (21), and (22) respectively represent the steam mass equation, vapor pressure equation, and average enthalpy of steam in the condenser. For the air zone, the governing equations are provided as [11, 12]:

$$\frac{dGa}{dt}=Gvb+Gn+Gg-Ga$$ (23)

$$\frac{dPa}{dt}=\frac{Ra(\frac{dGa}{dt})}{V}(Ts+273.15)$$ (24)

Pc=Ps+Pa (25)

where *Gvb*, *Gn*, *Gg*, *Ga*, *Pa*, *Ra*, and *Pc* respectively denote the air quantity of the condenser from the vacuum break valve, the air volume of the normal drain condense, the air amount from the seal leakage of the condenser, the air quantity from air extractor, the air pressure in the condenser, the gas constant of the air, and the absolute pressure. Eqs (23), (24), and (25) respectively represent air mass equation, air pressure equation, and absolute pressure equation in the condenser. For the hot water zone, the governing equations are provided as [11, 12]:

$$Lc=\frac{Gw}{\rho Aw}$$ (26)

$$\frac{dGw}{dt}=Gc+Ggp-Gwo$$ (27)

$$\frac{dGwHw}{dt}=Gc\times Hcw+Ggp\times Hgp-(Gwo\times Hw)$$ (28)

where *Lc*, *Gw*, *ρ*, *Aw*, *Ggp*, *Gwo*, *Hgp*, *Hw*, and *Hcw* respectively denote the hot well water level, the hot water quality, the hot well water density, the hot well cross-sectional area, the bubbling oxygen exhaust volume, the condenser water outlet quantity, the bubbling oxygen exhaust steam enthalpy, the enthalpy of hot well water, and enthalpy of saturated water corresponding to the condenser pressure. Eqs (26), (27), and (28) respectively represent hot well water level equation, hot water quality equation, and enthalpy equation of hot well water. The mathematical model of the condenser tube side is formulated by the dynamic heat balance equation of the circulating water, which is given by [11, 12]:

$$MwCw\frac{dT_{2}}{dt}=Q-Qw=UA\Delta tm-FcwCp(T-Tcw)$$ (29)

where *Mw*, *Cp*, Q, *Qw*, *U*, *Δtm*, *A*, *Fcw*, *Tcw*, and *T* respectively represent the circulating water quantity, the circulating water heat capacity, the steam outlet heat, the circulating water heat absorption quantity, the condenser heat transfer coefficient, the logarithmic mean temperature difference, the condenser heat transfer area, the circulating water flow, the circulating water inlet temperature, and the circulating water outlet temperature. The value of Δ*tm* is calculated as follows [11, 12].


$$\Delta tm=\frac{T-Tcw}{ln\;((Ts-Tcw)/(Ts-T))}$$ (30)

The overcooling of condenser is calculated by *Δtw* = *Tc*−*Tw* where *Tc* is the saturated water temperature of the vapor pressure in the condenser and Tw is the condenser hot well water temperature. The heat transfer error of the condenser is calculated by *δt* = *Ts*−*T* where *Ts* is saturated gas temperature corresponding to saturation pressure in condenser. In the context of the dynamic heat balance, we make the assumption that the total condensation is fixed and that the incoming steam and outgoing condensate are in a saturated state. Thus, the thermal energy transferred from steam to the flowing water is equivalent to the heat potential of the steam. The heat emitted by the steam may be estimated and computed using the following equations:

$$\frac{dT}{dt}=\frac{Fcw}{Mcw}(Tcw-T)+\frac{Q}{Mcw+Cp}$$ (31)

The mass balance of steam and condensate relies on the assumption of continuous space and constant volume of steam and air. To clarify, in order to sustain the desired amount of vapor condensation in the condenser (at a specific vacuum level), it is necessary to regulate the outflow of condensed water within a specific range. To simplify the model, we use the assumption that the condensate’s input and outflow are in a saturated state. Hence, the equation that represents the ideal gas model is:

$$\frac{dP}{dT}=\frac{RTc}{V}(Fs-Fc)$$ (32)

## FOPID controlled steam condenser system tuned by en-CSA

### Fundamentals of FOPID

The attainment of further enhancements is viable through the implementation of a FOPID controller, which constitutes a more sophisticated and generalized iteration of the conventional PID controller [43]. The superiority of FOPID control over PID control is evident across various dimensions [44]. Firstly, it integrates five specifications, affording two additional parameters compared to PID, thereby augmenting flexibility in control design. Secondly, it readily achieves iso-damping properties across a broader frequency range. Thirdly, it exhibits heightened robustness. Lastly, it yields superior outcomes for diverse scenarios, including higher-order systems, non-minimum phase systems, nonlinear systems, and systems characterized by extended time delays. The transfer function of a FOPID controller is expressed as follows, where *K*_*p*_, *K*_*i*_, *K*_*d*_, *λ*, and *μ* denote the proportional gain, integral gain, derivative gain, fractional integral order, and fractional derivative order, respectively [44].


$$C_{FOPID}(s)=K_{p}+\frac{K_{i}}{s^{\lambda }}+K_{d}s^{\mu }$$ (33)

**Fig 6:**
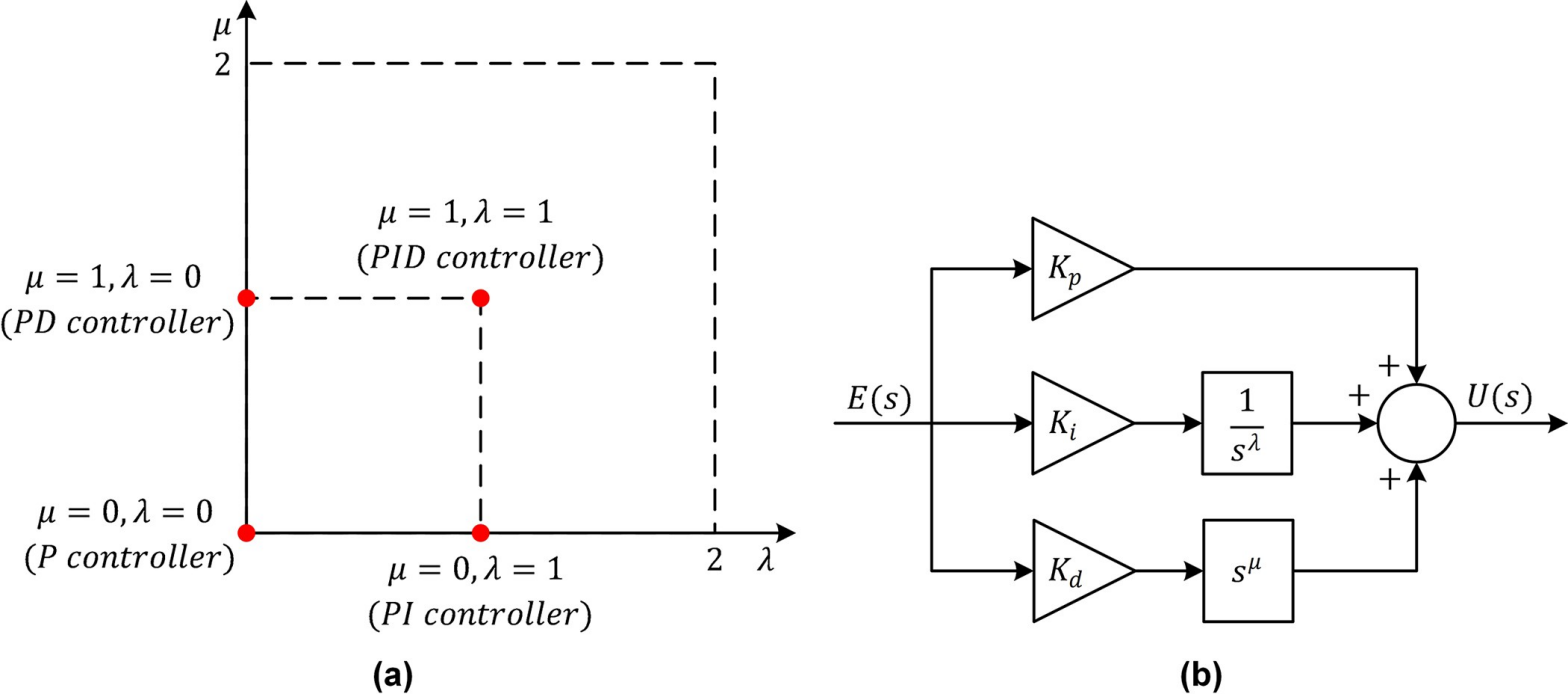
The plane (a) and block diagram (b) of a FOPID controller.

This equation reveals that the classical proportional (P), proportional-integral (PI), proportional-derivative (PD), and proportional-integral-derivative (PID) controllers can be discerned as specific instances of the FOPID controller, depending on the fractional order values. Fig 6(A) visually elucidates the correlation between FOPID and classical controllers, depicting the generalized FOPID control extending PID control from a singular point to a comprehensive plane. The associated block diagram of the FOPID controller is presented in Fig 6(B).

### Optimization problem and application of en-CSA

To ascertain a viable and effective solution for the speed condenser, the challenge is formulated as a constrained minimization problem, rendering it amenable to optimization algorithms. In outlining the minimization problem, this investigation employs a prescribed approach for optimizing the FOPID controller. Initially, the problem is expressed as $X=[x_{1},x_{2},x_{3},x_{4},x_{5}]=[K_{p},K_{i},K_{d},\lambda ,\mu ]$, and subsequently, the integral of time absolute error (ITAE) cost function [45] is embraced for judicious minimization using the proposed en-CSA.


$$ITAE=\int_{0}^{\infty }t\cdot |e(t)|\cdot dt$$ (34)

**Fig 7:**
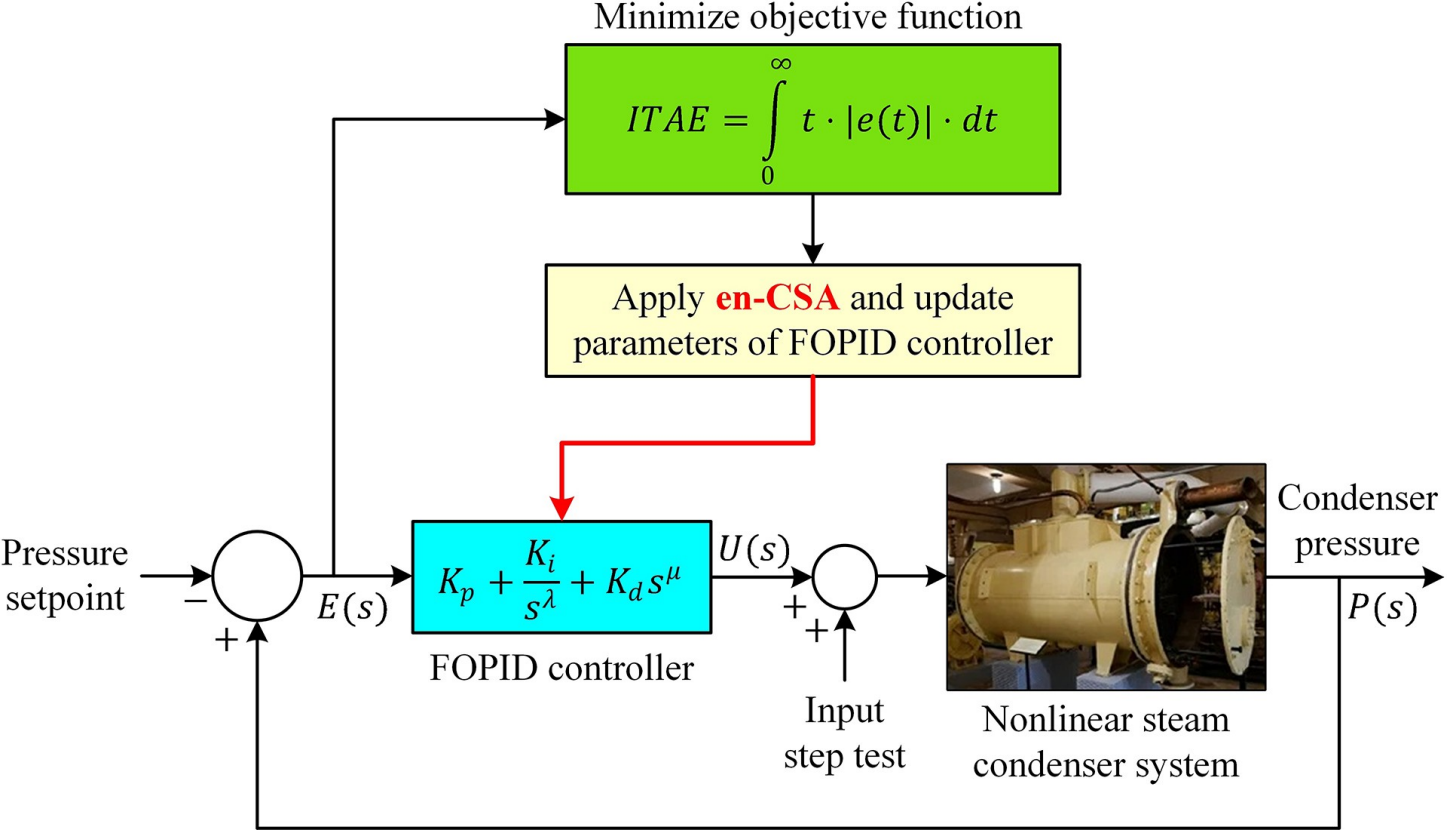
Application of the en-CSA to nonlinear condenser system.

Here, *e*(*t*) signifies the error signal, and the minimization of the ITAE cost function is constrained within the variable ranges: 1≤*K*_*p*_≤20, 0.1≤*K*_*i*_≤10, 0.05≤*K*_*d*_≤2, 0.5≤*λ*≤1.5 and 0.5≤*μ*≤1.5. Fig 7 elucidates the application of the en-CSA in the design of the FOPID-controlled nonlinear condenser system. In this context, the closed-loop response to a step change in the P setpoint, transitioning from 90 kPa to 95 kPa, is employed.

## Simulation results and discussion

### Parameters of test system

Before delving into the analyses and presenting the simulation results, it is imperative to establish a clear understanding of the parameters and variables governing the steam condenser system under examination. Table 4 provides a detailed compilation of these parameters and variables, each crucial for the accurate representation and evaluation of the system’s performance. The values assigned to parameters are documented in the table, expressed in their respective units.

**Table 4:** Parameters/variables of steam condenser system.

Parameter/Variable	Value	Unit
*R*	0.461526	kJ/kgK
*V*	3	m^3^
*λ*	2265.65	kJ/kg
*UA*	356.972	kW/K
*Mcw*	6500	kg
*Cp*	4.2	kJ/(kgK)
*α*	0.3162	K/kPa
*β*	68.0958	°C
*α*_1_	0.087292	-
*α*_2_	0.00073787	-
*Fs*	4	kg/s
*Fc*	4	kg/s
*Fcw*	107.8881	kg/s
*P*	90	kPa
*T*	80	°C
*Tcw*	60	°C
*Tc*	96.5538	°C
*Q*	9062.6	kW

From a scientific standpoint, these parameters include key factors such as the heat transfer coefficients, steam flow rates, condenser pressure, and cooling water temperature, among others. Each of these parameters plays a significant role in determining the dynamic behavior and performance of the steam condenser system. For instance, the heat transfer coefficient directly influences the efficiency of heat exchange between the steam and cooling water, impacting the overall thermal performance of the condenser.

Analyzing from a physical perspective, understanding these parameters is vital for accurately simulating the system’s response to various control strategies. The heat transfer coefficient, for example, determines how effectively the steam condenses and transfers heat to the cooling water. Variations in steam flow rates affect the pressure and temperature within the condenser, influencing the condensation process and the system’s thermal efficiency. Cooling water temperature, another critical parameter, impacts the condenser’s ability to maintain optimal pressure and temperature levels for efficient operation.

Furthermore, the interplay between these parameters needs to be thoroughly understood to develop and validate effective control strategies. The FOPID controller, optimized using the en-CSA, relies on accurate modeling of these parameters to achieve precise control of the condenser pressure. Any deviations or inaccuracies in these parameters can significantly affect the controller’s performance, leading to suboptimal pressure regulation and potential system instability.

Thus, the detailed compilation of these parameters in Table 4 serves as the foundation for the subsequent simulation analyses. By accurately defining and understanding these variables, we can ensure that the simulation results are reflective of the real-world behavior of the steam condenser system. This, in turn, allows for a more reliable evaluation of the en-CSA optimized FOPID controller’s effectiveness in managing the condenser pressure and enhancing overall system performance.

### Statistical performance of recommended en-CSA

In order to evaluate the suggested en-CSA, this study compared it to three other modern and effective optimization algorithms: the Runge-Kutta optimizer (RUN) [40], prairie dog optimization (PDO) [41] and RIME optimizer [42]. A fair assessment was conducted with a population size of 30 and a total iteration number of 50, repeated 25 times to ensure robustness and reliability of the results.

From a scientific perspective, this comparative analysis is essential to ascertain the efficacy and robustness of en-CSA in solving optimization problems. The choice of these benchmark algorithms provides a diverse set of optimization strategies, allowing for a comprehensive evaluation of en-CSA’s performance across different paradigms.

**Fig 8:**
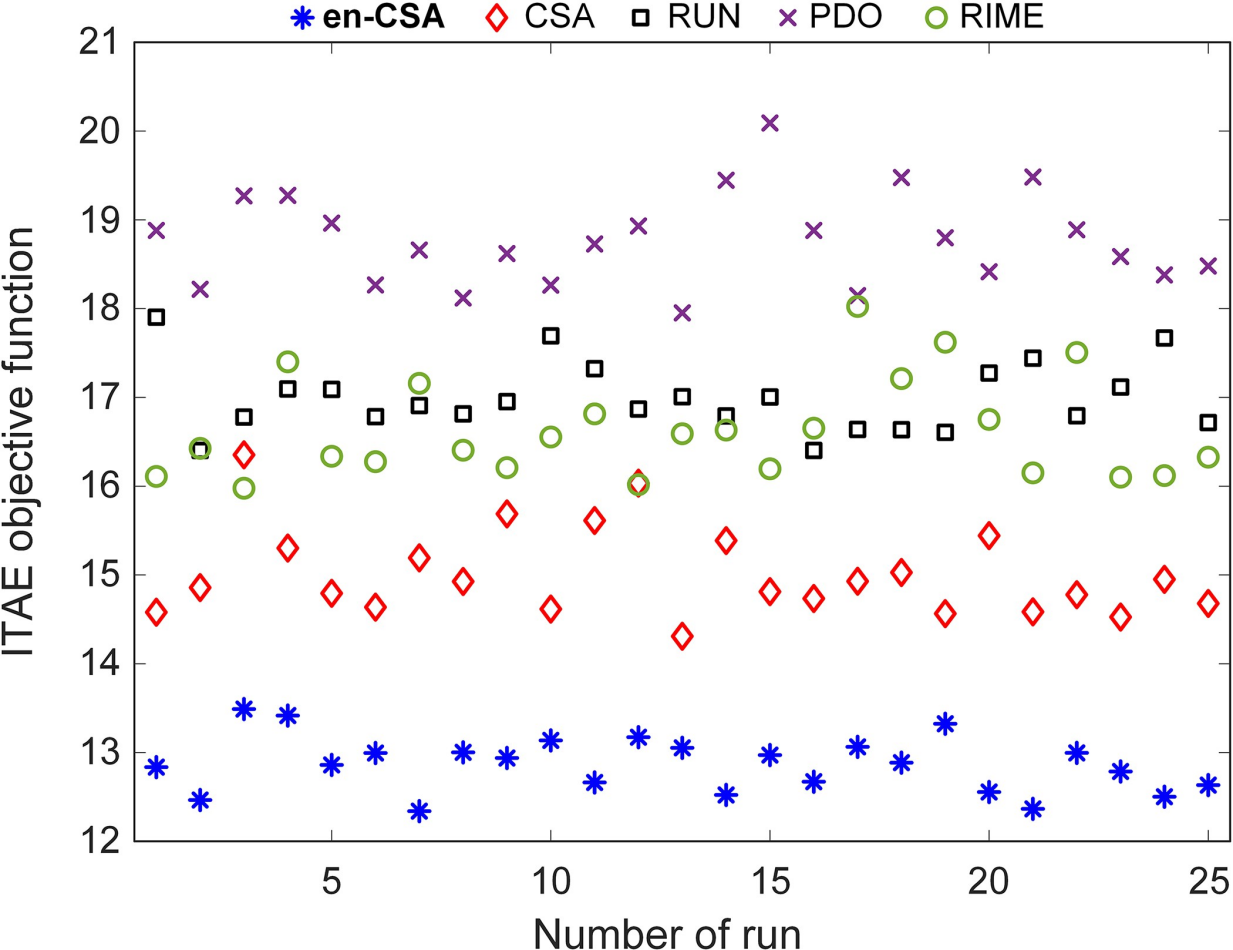
ITAE objective function values for all runs of en-CSA, CSA, RUN, PDO and RIME algorithms.

Fig 8 illustrates the integral of time-weighted absolute error (ITAE) objective function values for all runs. It is clear that en-CSA consistently achieves the lowest values with the smallest standard deviation, indicating its superior optimization capability. The ITAE metric, commonly used in control systems, measures the performance by integrating the absolute error over time, weighted by time. Lower ITAE values signify better performance, as they indicate quicker and more accurate convergence to the desired solution.

**Fig 9:**
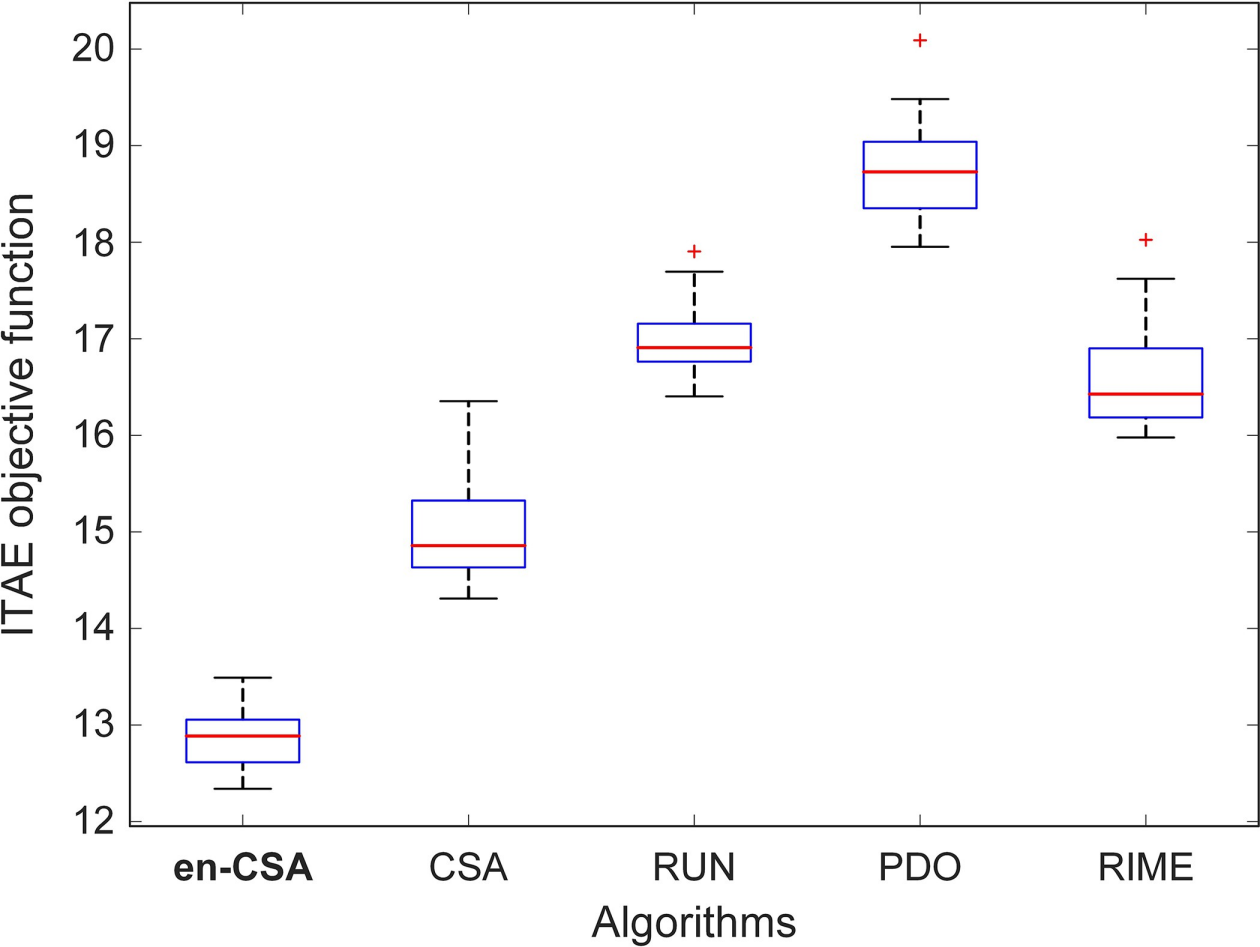
Boxplot analysis for ITAE objective function using en-CSA, CSA, RUN, PDO and RIME algorithms.

Furthermore, Fig 9 presents a boxplot analysis, reaffirming that en-CSA attains the lowest objective function values with a comparatively small distribution. Boxplots visually summarize the distribution, central tendency, and variability of the data, highlighting the consistency and reliability of en-CSA’s performance. The narrow spread of the en-CSA boxplot, coupled with its low median values, underscores its robust performance in achieving optimal solutions.

To provide a quantitative comparison, Table 5 presents numerical statistical analyses across various metrics, including the best, worst, mean, and standard deviation (SD) values. Notably, en-CSA outperforms the other algorithms in terms of the best, worst, and mean values, with the lowest ITAE values recorded. The standard deviation for en-CSA is also remarkably lower than its counterparts, indicating a higher degree of consistency and stability in its performance. From a physical perspective, this stability is crucial as it implies that en-CSA can reliably find optimal solutions across multiple runs, a desirable trait for practical applications where consistency is key.

**Table 5:** Comparative numerical statistical analysis.

Metric	en-CSA	CSA	RUN	PDO	RIME
Best	12.3398	14.3100	16.4032	17.9525	15.9775
Worst	13.4899	16.3535	17.9041	20.0906	18.0242
Mean	12.8661	15.0131	16.9889	18.7688	16.6231
SD	0.3154	0.5031	0.3875	0.5221	0.5614

These findings collectively support the assertion that the recommended en-CSA algorithm demonstrates superior statistical performance when compared to the selected state-of-the-art optimization algorithms. The enhanced mechanisms within en-CSA, such as the control randomization operator and adaptive p-best mutation, contribute significantly to its ability to explore and exploit the search space effectively, thereby achieving better optimization outcomes.

### Evolution of ITAE and best FOPID parameters

**Fig 10:**
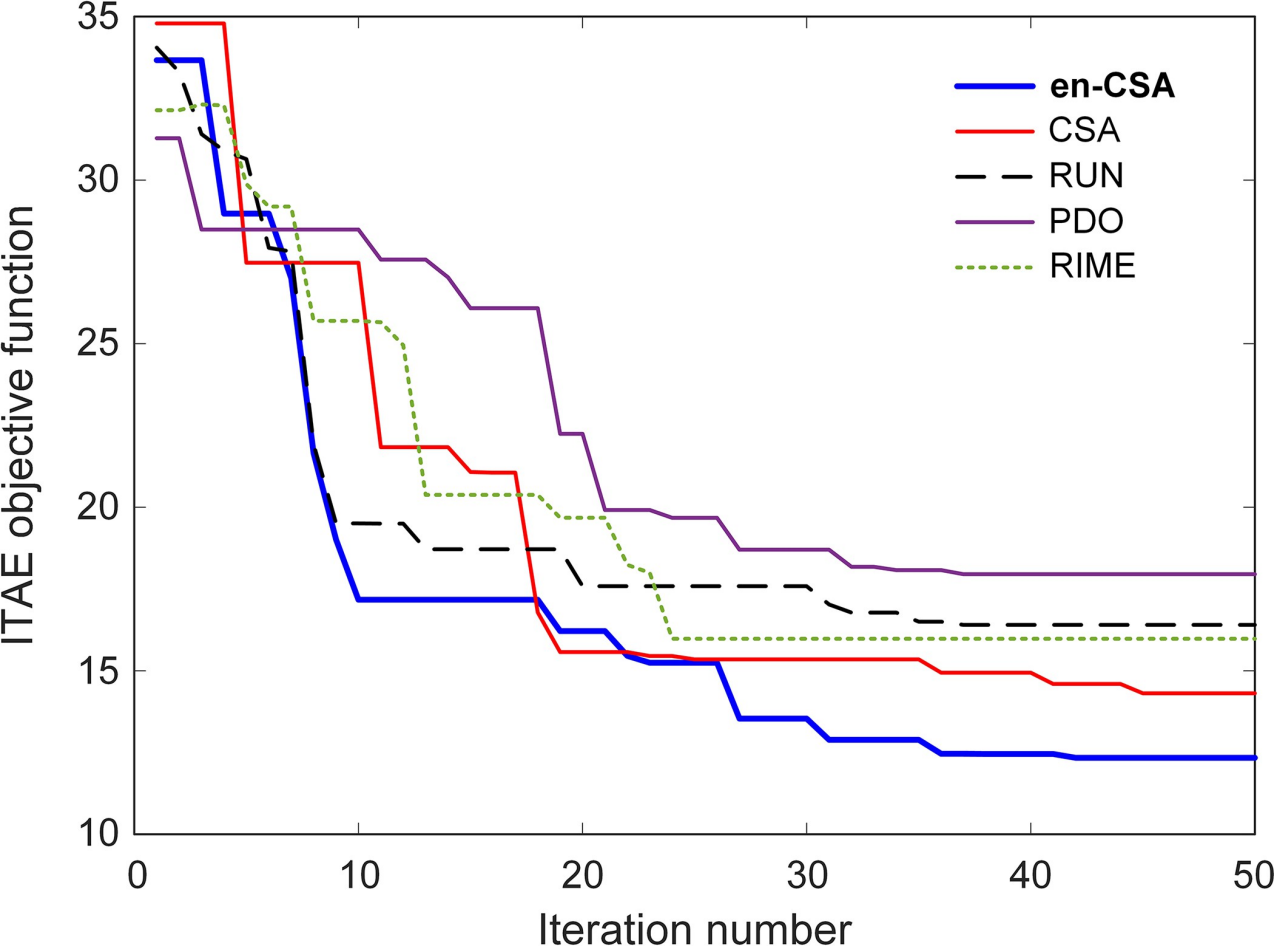
Change of ITAE objective function for en-CSA, CSA, RUN, PDO and RIME algorithms.

Fig 10 visually captures the dynamic changes in the ITAE objective function, emphasizing the evolution of ITAE and the determination of optimal FOPID parameters. Metaheuristic optimization methods, characterized by their swarm-based nature, navigate solution spaces randomly to seek optimal solutions. These methods aim to minimize the ITAE objective function within the confines of the solution space during each iteration, ultimately converging toward the best possible solutions.

From a scientific standpoint, ITAE is a crucial performance metric for control systems, as it combines both the magnitude of the error and the duration for which the error persists. Lower ITAE values indicate a more effective control system, as the system quickly and accurately responds to disturbances.

Fig 10 illustrates the fluctuations of the ITAE objective function across specified iterations for all algorithms, each displaying distinct convergence curves based on their search mechanism characteristics. The en-CSA algorithm is particularly distinguished by its well-balanced mechanism, which allows it to achieve global solutions without being affected by local minima or premature convergence, unlike other algorithms. This balanced approach is essential in maintaining diversity in the solution space, preventing the algorithm from settling prematurely on suboptimal solutions.

Physically, en-CSA’s rapid convergence to lower ITAE values in early iterations indicates its efficiency in exploring the solution space and identifying optimal FOPID controller parameters. The algorithm’s ability to maintain a broad search early on, followed by a more focused exploitation phase, enables it to fine-tune the FOPID parameters effectively. This swift convergence underscores the efficiency and effectiveness of en-CSA in optimizing control parameters, ensuring that the FOPID controller performs optimally under varying conditions.

Additionally, Table 6 supplements the visual representation by detailing the obtained controller parameters through different algorithms. The comparative analysis of FOPID parameters obtained by en-CSA and other algorithms offers deeper insights into the optimization process. The table showcases the specific values for the FOPID controller’s parameters (e.g., proportional gain, integral gain, derivative gain, and fractional orders) optimized by each algorithm, providing a quantitative basis for comparing their performance.

**Table 6:** Obtained controller parameters via different algorithms.

Parameters	en-CSA	CSA	RUN	PDO	RIME
*K*_*p*_	5.5547	5.2449	5.8877	5.2479	5.7250
*K*_*i*_	0.9190	0.8503	0.8810	1.4482	1.1871
*K*_*d*_	1.9675	1.2131	1.3916	1.9420	1.7104
*λ*	1.0005	1.0022	1.0021	0.9943	0.9958
*μ*	0.9332	0.9157	0.8746	0.7363	0.8054

By examining these parameters, one can gain a deeper understanding of how en-CSA fine-tunes the controller settings to achieve superior performance. For instance, the en-CSA may adjust the proportional gain to ensure a swift response to errors, while simultaneously fine-tuning the integral and derivative gains to minimize steady-state error and dampen oscillations, respectively. The fractional orders in the FOPID controller allow for a more flexible and precise adjustment of the control action, further enhancing the system’s performance.

### Nonlinear simulation result

**Fig 11:**
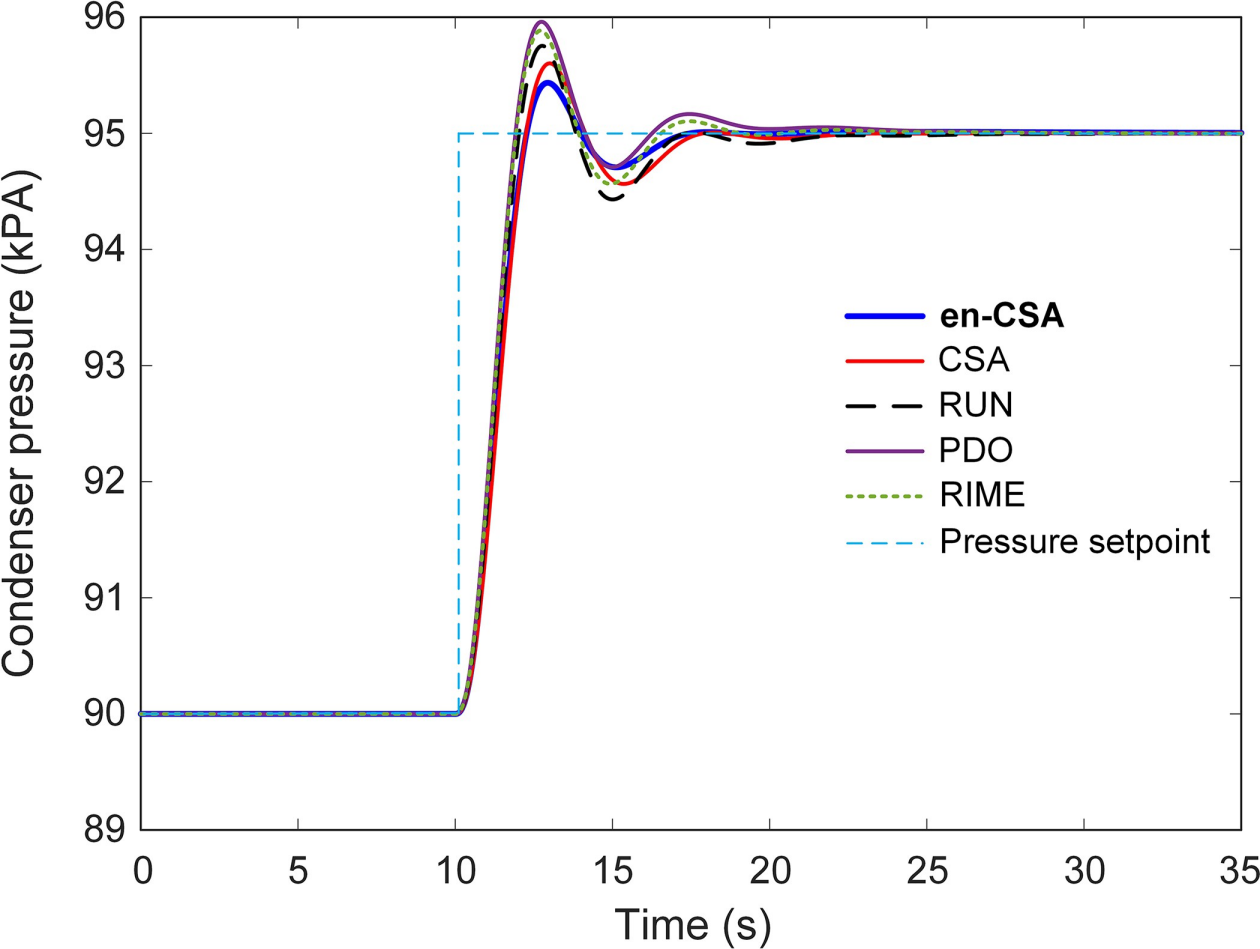
Time response of the en-CSA based condenser with respect to different algorithms of CSA, RUN, PDO and RIME.

**Fig 12:**
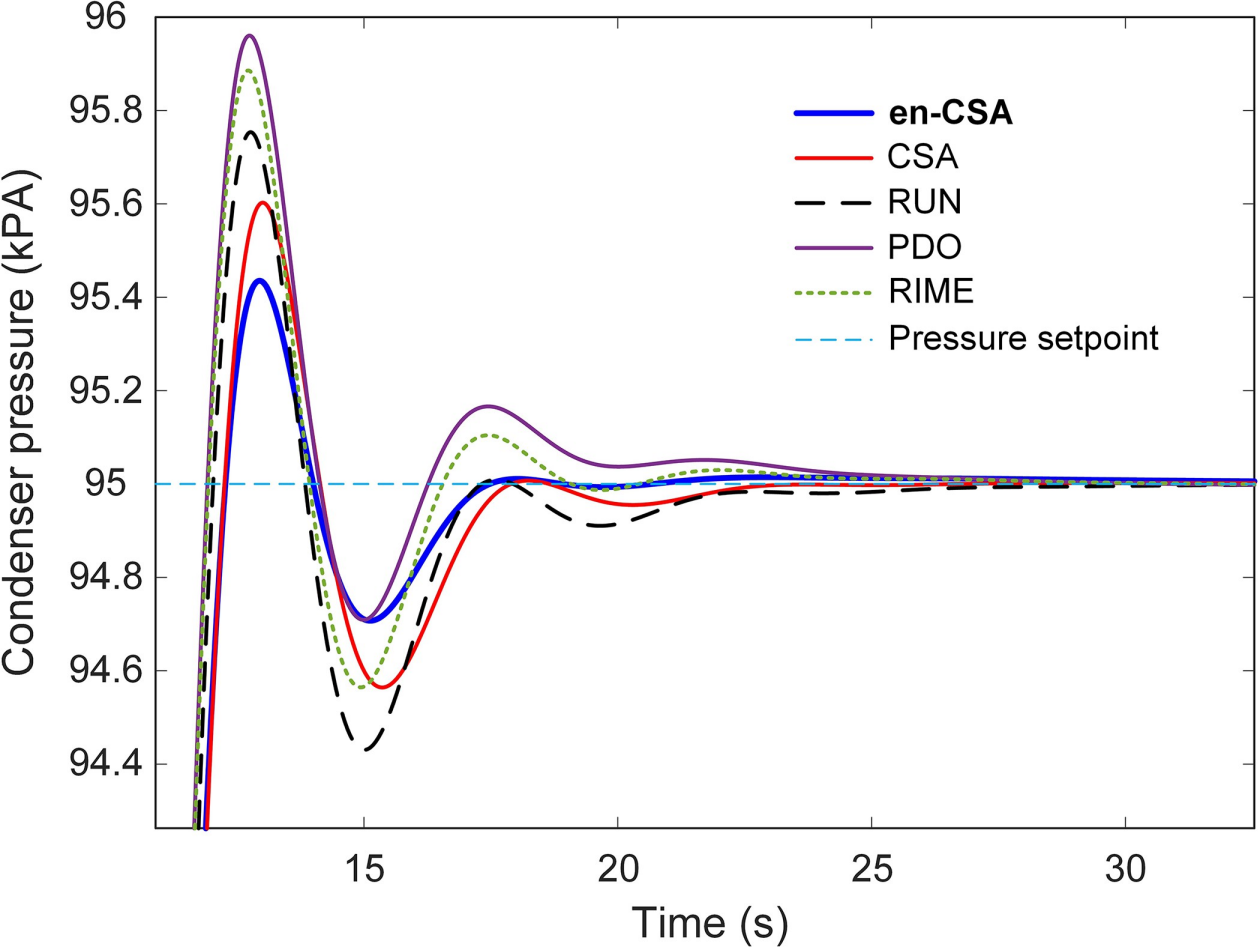
Zoomed view of Fig 11.

In the examination of nonlinear simulation results, Fig 11 illustrates the time response of the condenser under the influence of various optimization algorithms, each contributing to the evolution of the system’s behavior. The system undergoes a step change in setpoint pressure, transitioning from 90 kPA to 95 kPA at the 10-second mark. This dynamic change is further elucidated in Fig 12, which provides a zoomed-in view of Fig 11, facilitating a detailed inspection of the system’s response to the altered setpoint.

From a scientific standpoint, analyzing the time response of the condenser is crucial for understanding how well the control algorithms manage sudden changes in operating conditions. The step change in setpoint pressure simulates a realistic scenario where the system must quickly adapt to new conditions, reflecting its robustness and adaptability.

The efficacy of the recommended en-CSA becomes particularly evident in Fig 11, where it exhibits the lowest overshoot and settling time compared to other algorithms. Overshoot and settling time are critical parameters in control systems. Overshoot refers to the extent to which the system exceeds its target value, while settling time is the duration the system takes to stabilize within a certain range around the target value. Minimizing these parameters is essential for achieving a stable and efficient control system.

**Fig 13:**
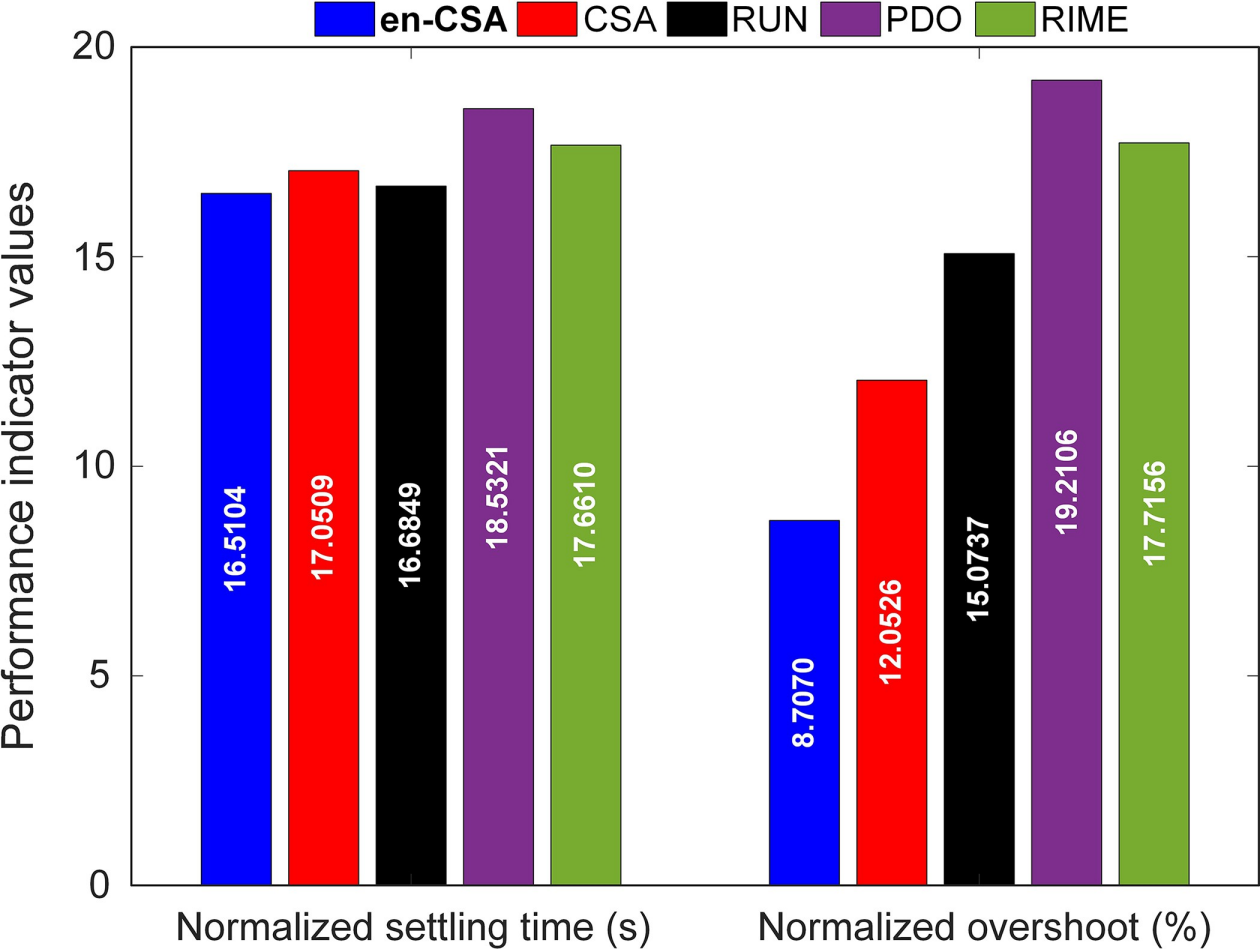
Comparison of normalized settling times and overshoots for en-CSA, CSA, RUN, PDO and RIME methods.

This observation is further quantified in Fig 13, which offers a comparative analysis of normalized settling times and overshoots for en-CSA, alongside other algorithms such as CSA, RUN, PDO, and RIME. The normalized metrics provide a standardized way to compare the performance across different algorithms, making the analysis more robust and generalizable.

Especially, en-CSA demonstrates a normalized settling time of 16.5104 and a normalized overshoot of 8.7070%, establishing it as the algorithm with the most favorable performance in minimizing overshoot and achieving a swift settling time. These outcomes underscore the effectiveness of en-CSA in optimizing the condenser system’s time response dynamics, thereby contributing to enhanced control and stability.

Physically, the reduced overshoot achieved by en-CSA means that the system can reach its new setpoint without excessive fluctuations, which is crucial for maintaining the integrity and efficiency of industrial processes. A swift settling time indicates that the system can quickly stabilize after a disturbance, ensuring minimal downtime and consistent performance.

The superiority of en-CSA can be attributed to its balanced exploration-exploitation mechanism, which allows it to efficiently navigate the solution space and identify optimal control parameters. By maintaining high population diversity and employing adaptive strategies, en-CSA effectively avoids local optima and ensures robust convergence to the global optimum.

### Comparison with published approaches

**Fig 14:**
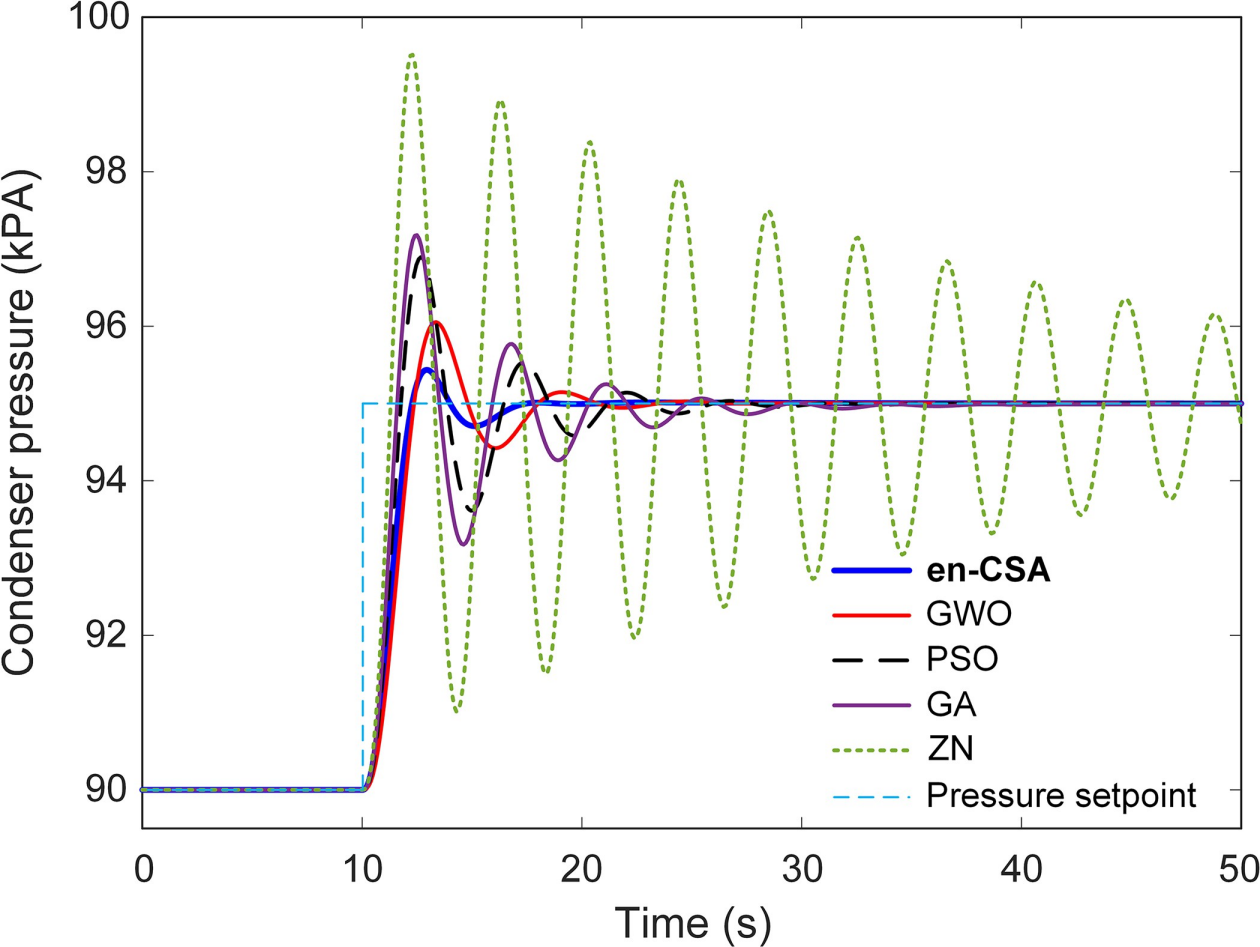
Time response of the en-CSA based condenser with respect to reported approaches of GWO, PSO, GA and ZN.

In the context of comparing the performance of the recommended en-CSA with established approaches reported in the literature, this section employs the time response analysis and subsequent metrics for evaluation. Fig 14 displays the time-dependent response of the condenser system when it experiences a sudden shift in setpoint pressure, going from 90 kPA to 95 kPA at the 10-second mark. It is worth mentioning that en-CSA shows the smallest amount of overshoot and takes the least amount of time to settle in this dynamic situation, as clearly shown in the depiction.

**Fig 15:**
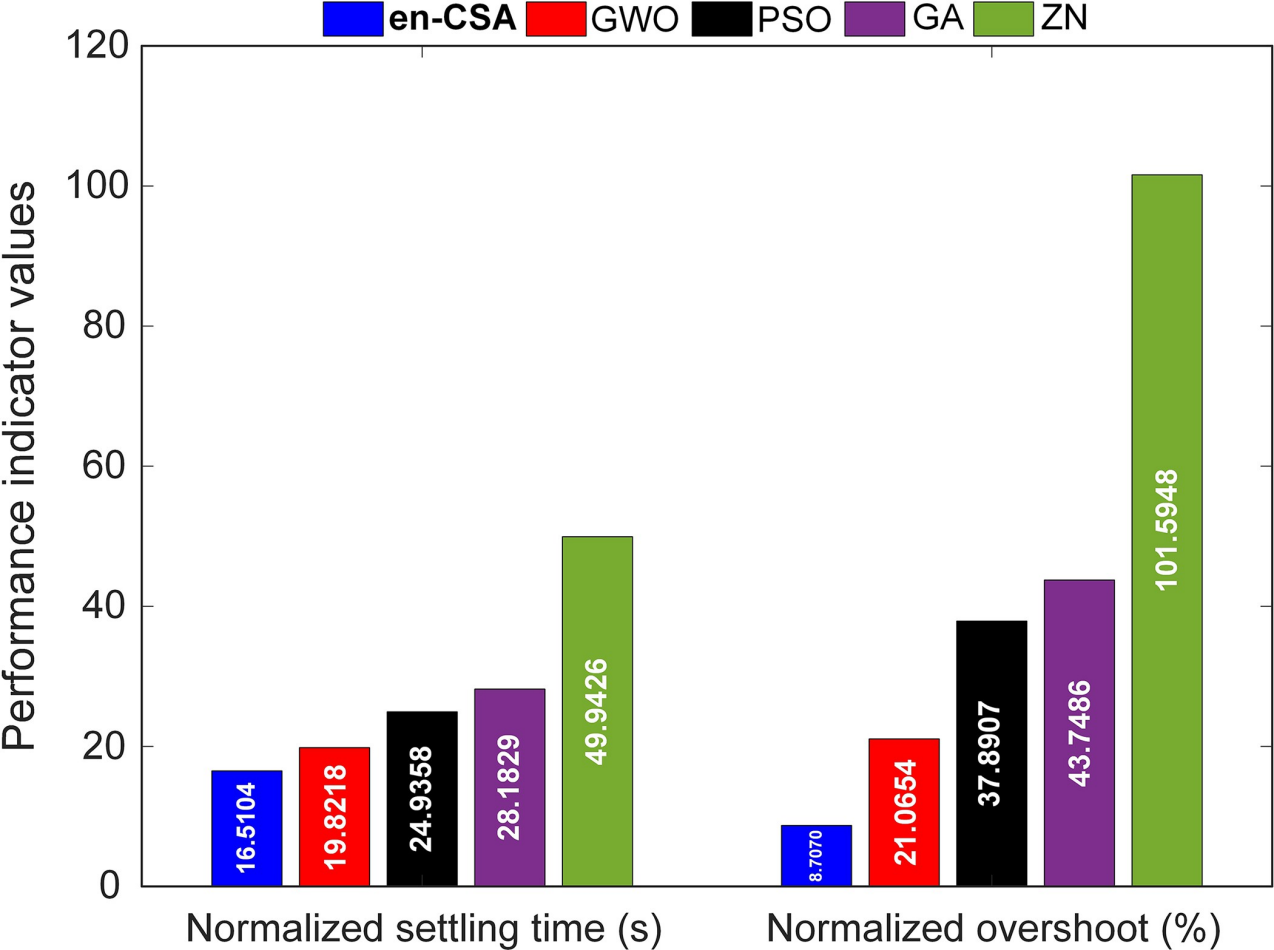
Comparison of normalized settling times and overshoots for en-CSA, GWO, PSO, GA and ZN methods.

Fig 15 presents a quantitative evaluation of normalized settling times and overshoots for en-CSA, compared to other methods such as grey wolf optimizer (GWO), particle swarm optimization (PSO), genetic algorithm (GA), and Ziegler-Nichols (ZN) [11]. The en-CSA is remarkable for its normalized settling time of 16.5104 seconds and a normalized overshoot of 8.7070%, which are the lowest values compared to other optimization methods. This result demonstrates the exceptional effectiveness of en-CSA in delivering a quick and consistent response, exceeding the performance of previously documented methods in the literature.

## Conclusion

This study addresses the critical need for optimizing shell and tube heat exchangers in industrial processes, particularly for pressure regulation. For the control aspect, we used the FOPID controller due to its promising feature of capturing the dynamic behavior of complex systems. The novel contribution of this work lies in the introduction and application of en-CSA optimizer for tuning the FOPID controller. The innovative features of en-CSA include a control randomization operator, linear transfer function, and adaptive p-best mutation integrated with original CSA. The effectiveness of en-CSA was rigorously tested using the CEC2020 benchmark functions, demonstrating superior convergence rates and accuracy in locating optimal solutions compared to other optimization algorithms. Application of en-CSA to FOPID controller tuning in shell and tube heat exchangers yielded remarkable results, with significant improvements in pressure regulation, faster response times (up to 11%), and minimized overshooting (up to 55%) compared to alternative methods. These findings underscore en-CSA’s potential as a reliable tool for optimizing control parameters in real-world scenarios, thereby enhancing operational stability and efficiency in industrial processes. The success of en-CSA in both benchmark functions and practical steam condenser control highlights its versatility and broad applicability as a powerful optimization tool.

## List of abbreviations

CEC2020: IEEE Congress on Evolutionary Computation 2020
CGO: Chaos Game Optimization
CSA: Cooperation Search Algorithm
en-CSA: Enhanced Cooperation Search Algorithm
FOPID: Fractional-Order Proportional-Integral-Derivative
GA: Genetic Algorithm
GTO: Giant Trevally Optimizer
GWO: Grey Wolf Optimizer
HHO: Harris Hawks Optimization
ITAE: Integral of Time Absolute Error
LTF: Linear Transfer Function
PDO: Prairie Dog Optimization
PI: Proportional-Integral
PID: Proportional-Integral-Derivative
PSO: Particle Swarm Optimization
RUN: Runge-Kutta Optimizer
SSA: Salp Swarm Algorithm
TSA: Tree-Seed Algorithm
WOA: Whale Optimization Algorithm
ZN: Ziegler-Nichols
R: Gas constant
V: Volume
λ: Latent heat
UA: Overall heat transfer coefficient
Mcw: Mass of cooling water
Cp: Specific heat capacity
α: Temperature-pressure coefficient
β: Temperature coefficient
*α*_1_: Coefficient (dimensionless)
*α*_2_: Coefficient (dimensionless)
Fs: Steam flow rate
Fc: Cooling water flow rate
Fcw: Flow rate of cooling water
P: Pressure
T: Temperature
Tcw: Cooling water temperature
Tc: Condenser temperature
Q: Heat transfer rate
